# Catalyst–Support Interaction in Polyaniline-Supported Ni_3_Fe Oxide to Boost Oxygen Evolution Activities for Rechargeable Zn-Air Batteries

**DOI:** 10.1007/s40820-024-01511-4

**Published:** 2024-09-21

**Authors:** Xiaohong Zou, Qian Lu, Mingcong Tang, Jie Wu, Kouer Zhang, Wenzhi Li, Yunxia Hu, Xiaomin Xu, Xiao Zhang, Zongping Shao, Liang An

**Affiliations:** 1 Department of Mechanical Engineering, The Hong Kong Polytechnic University, Hung Hom, Kowloon, Hong Kong SAR, People’s Republic of China; 2 Jiangsu Key Laboratory of Atmospheric Environment Monitoring and Pollution Control, Jiangsu Collaborative Innovation Center of Atmospheric Environment and Equipment Technology, School of Environmental Science and Technology, Nanjing University of Information Science and Technology, Nanjing, 210044 People’s Republic of China; 3 Department of Chemistry, The Chinese University of Hong Kong, Ma Lin Building, Shatin, Hong Kong SAR, 999077 People’s Republic of China; 4 WA School of Mines: Minerals, Energy and Chemical Engineering (WASM-MECE), Curtin University, Perth, WA 6102 Australia; 5 Research Institute for Advanced Manufacturing, The Hong Kong Polytechnic University, Hung Hom, Kowloon, Hong Kong SAR, People’s Republic of China; 6 Research Institute for Smart Energy, The Hong Kong Polytechnic University, Hung Hom, Kowloon, Hong Kong SAR, People’s Republic of China

**Keywords:** Catalyst–support interaction, Supported catalysts, Heterointerface, Oxygen evolution reaction, Zn-air batteries

## Abstract

Catalyst–support interaction plays a crucial role in improving the catalytic activity of oxygen evolution reaction (OER). Here we modulate the catalyst–support interaction in polyaniline-supported Ni_3_Fe oxide (Ni_3_Fe oxide/PANI) with a robust hetero-interface, which significantly improves oxygen evolution activities with an overpotential of 270 mV at 10 mA cm^−2^ and specific activity of 2.08 mA cm_ECSA_^−2^ at overpotential of 300 mV, 3.84-fold that of Ni_3_Fe oxide. It is revealed that the catalyst–support interaction between Ni_3_Fe oxide and PANI support enhances the Ni–O covalency via the interfacial Ni–N bond, thus promoting the charge and mass transfer on Ni_3_Fe oxide. Considering the excellent activity and stability, rechargeable Zn-air batteries with optimum Ni_3_Fe oxide/PANI are assembled, delivering a low charge voltage of 1.95 V to cycle for 400 h at 10 mA cm^−2^. The regulation of the effect of catalyst–support interaction on catalytic activity provides new possibilities for the future design of highly efficient OER catalysts.

**Supplementary Information:**

The online version contains supplementary material available at 10.1007/s40820-024-01511-4.

## Highlights

- Ni_3_Fe oxide, with an average size of 3.5 ± 1.5 nm, was successfully deposited onto polyaniline (PANI) support through a solvothermal strategy followed by calcination.
- The catalyst–support interaction between Ni_3_Fe oxide and PANI can enhance the Ni-O covalency via the interfacial Ni-N bond.
- Ni_3_Fe oxide/PANI-assembled Zn-air batteries achieve superior cycling life for over 400 h at 10 mA cm^−2^ and a low charge potential of around 1.95 V.

**Supplementary Information:**

The online version contains supplementary material available at 10.1007/s40820-024-01511-4.

## Graphical abstract

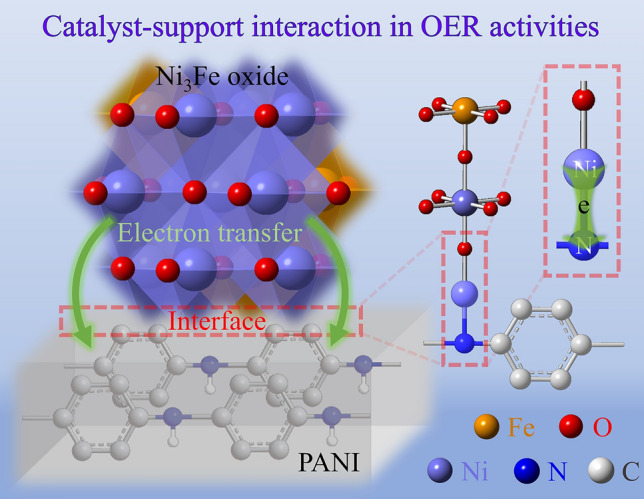

## Introduction

Rechargeable Zn-air batteries have attracted much attention as the next-generation energy systems considering their high theoretical energy density of 1086 Wh kg^−1^, environmentally friendliness, low cost, and high safety [1–3]. Unfortunately, rechargeable Zn-air batteries suffer from sluggish kinetics of oxygen evolution reaction (OER) in air cathode, thus inducing high charge overpotential and poor charge rate [4–6]. Although noble metal Ru or Ir-based catalysts exhibit acceptable OER activity, the poor stability and high cost of noble metals have hindered their practical application in Zn-air batteries [7, 8]. Therefore, there is an urgent need to search for highly efficient OER catalysts with low cost, high catalytic activity, and excellent electrochemical stability.

To date, great efforts have been devoted to pursuing transition metal compounds, including metal oxides [9, 10], layered double hydroxides [11], nitrides [12], sulfides [13], etc., as OER electrocatalysts in lowering the charge potential for Zn-air batteries. Nitrides and sulfides would undergo severe electrochemical reconstruction, causing a serious crystal structure change, thus greatly affecting their stability and activity [14]. Differently, metal oxides exhibit better crystal structure stability during OER process, adjustable crystal structure, and ease of synthesis, which makes them promising OER catalyst candidates in practical devices [15, 16]. Especially, the spinel oxides with the formula of AB_2_O_4_ (A, B = transition metal) attached with rich redox couples (A^3+^/A^2+^ and B^3+^/B^2+^) have achieved considerable attention owing to adjustable composition and geometrical configuration [17–20]. Among them, NiFe-based spinel oxides are considered as a desirable OER catalyst benefiting from the advantage of high OER activity and low cost of Ni and Fe elements [21]. However, the low conductivity and easy aggregation of NiFe oxides affect electron conduction and mass transfer rate [22]. The preferable strategy currently reported is to introduce porous conductive supports to anchor NiFe oxides, which could improve the dispersion of catalyst nanoparticles to enhance the density of catalytic active sites and inhibit the electrochemical aggregation of catalyst nanoparticles during the OER process [23, 24]. Searching for suitable support and understanding the catalyst–support interaction are crucial for regulating the OER activity and stability of NiFe oxides.

Porous carbon, such as heteroatom-doped carbon nanotube [25], graphene [26], and porous carbon [27], is currently the most widely used support for loading metal oxide catalysts in OER. However, the defective carbon caused by heteroatom doping is prone to electrochemical corrosion during the OER process, thus destroying the supported structure. Besides, the introduction of defect sites in carbon supports usually requires the treatment by strong acid [28, 29]. In contrast, conductive polymers exhibit high conductivity, excellent electrochemical stability, facile structural adjustability, and abundant anchoring sites, which are considered a suitable candidate to replace carbon supports [30, 31]. Among many conductive polymers, polyaniline (PANI) has attracted great attention in electrocatalysis due to its unique π-conjugated structures, enriched nitrogen sites, and favorable hydrophilic properties [32, 33]. For instance, Yang et al. designed a PANI-supported NiSe nanoparticle (NiSe–PANI) catalyst for OER. The electron delocalization between Ni d-orbitals and PANI π-conjugated ligands induces a reasonable electron transfer from NiSe catalysts to PANI supports, thus promoting the OER activity and stability [34]. Given the above discussion, excellent OER catalytic activity may be achieved by using PANI supports to load NiFe oxides meanwhile adjusting the catalyst–support interaction. However, the mechanism by which the catalyst–support interaction regulates OER catalytic activity in polyaniline-supported Ni_3_Fe oxide is still lacking in understanding.

Herein, we designed a supported catalyst by supporting Ni_3_Fe oxide, with an average size of 3.5 ± 1.5 nm, on PANI support through a solvothermal strategy followed by calcination. The catalyst–support interaction between Ni_3_Fe oxide and PANI can enhance the Ni–O covalency via the interfacial Ni–N bond. We further find that the PANI support can promote the charge, electron, and mass transfer on Ni_3_Fe oxide, thus optimizing the OER catalytic activity of Ni_3_Fe oxide. As a result, the Ni_3_Fe oxide/PANI catalyst delivers a low OER overpotential of 270 mV at 10 mA cm^−2^, a small Tafel slope of 60 mV dec^−1^, and a long lifetime of 150 h at 10 mA cm^−2^, surpassing the Ni_3_Fe oxide alone and the commercial IrO_2_ standard. Besides, Ni_3_Fe oxide/PANI-assembled Zn-air batteries achieve a superior cycling life for over 400 h at 10 mA cm^−2^ and a low charge potential of around 1.95 V. We believe this work offers a new perspective on understanding the effect of catalyst–support interaction in enhancing OER catalytic activity.

## Experimental Section

### Materials

The gas diffusion layer was bought from Changzhou Youteke New Energy Technology Co., Ltd (China). NiCl_2_·6H_2_O (AR, 98.5%) and FeCl_2_·4H_2_O (AR, 98.5%) were purchased from Macklin. Hexamethylenetetramine (AR, 99.0%) was gained from Sinopharm Chemical Reagent Co., Ltd (China). Dimethylformamide (AR, 99.5%), KOH (GR, 99.99%), and Zn(Ac)_2_ (AR, 99.5%) were acquired from Aladdin Co., Ltd (China). All reagents were employed without further purification.

### Catalyst Synthesis

Ni_3_Fe oxide/PANI and control catalysts were prepared through a solvothermal method followed by a calcination process in air. In detail, 0.05 g commercial PANI was firstly dispersed into 30 mL N, N-dimethylformamide (DMF) through ultrasonic treatment for 30 min. Then, 0.056 g FeCl_2_·4H_2_O and 0.2 g NiCl_2_·6H_2_O were dissolved into above solution by ultrasonication for 10 min. Finally, 0.2 g hexamethylenetetramine was dissolved into the obtained solution via ultrasonic treatment again. Subsequently, the mixed solution was sealed into a 50 mL hydrothermal autoclave reactor and further treated for 5 h at 160 °C in a bake oven. After cooled to room temperature, the catalyst precursor was collected via vacuum filtration and washed with deionized water and methanol several times. Further, the catalyst precursor was dried for 3 h at 80 °C in a bake oven. Finally, the dried catalyst precursor was annealed at 350 °C in a muffle furnace for 3 h with a heating rate of 5 °C min^−1^ to obtain Ni_3_Fe oxide/PANI catalysts. For comparison, Ni_3_Fe oxide catalysts were also prepared through a similar preparation procedure as Ni_3_Fe oxide/PANI catalysts without adding PANI. In addition, Ni oxide/PANI and Fe oxide/PANI catalysts were synthesized through the same preparation procedure as Ni_3_Fe oxide/PANI catalysts with just adding 0.267 g NiCl_2_·6H_2_O and 0.224 g FeCl_2_·4H_2_O, respectively, into the PANI-DMF solution. To study the effect of calcination in air, Ni_3_Fe oxide/PANI, Ni_3_Fe oxide, Ni oxide/PANI, and Fe oxide/PANI were also prepared through a solvothermal method without following the calcination process in air. To explore the optimal ratio of Ni and Fe, Ni_3.5_Fe_0.5_ oxide/PANI, Ni_2.5_Fe_1.5_ oxide/PANI, and Ni_2_Fe_2_ oxide/PANI catalysts were fabricated through a solvothermal method followed by an calcination process similar to Ni_3_Fe oxide/PANI, while the molar ratio of Ni: Fe was set to 3.5: 0.5, 2.5: 1.5, and 2: 2, respectively, and the mass loading of catalysts, Ni_3.5_Fe_0.5_ oxide, Ni_2.5_Fe_1.5_ oxide, and Ni_2_Fe_2_ oxide, on PANI was kept consistent with that of Ni_3_Fe oxide on PANI. Moreover, to explore the function of air calcination procedure for the designed catalysts, we prepare the Ni_3_Fe oxide/PANI-without calcination, Ni oxide/PANI-without calcination, and Fe oxide/PANI-without calcination catalysts by adding the Ni or Fe-based precursors with hexamethylenetetramine into DMF solvent and then treated for 5 h at 160 °C in sealed autoclave reactor as the above experiments; finally, the obtained catalyst powder was collected via vacuum filtration and washed with deionized water and methanol several times.

## Results and Discussion

### Characterization of Morphology

**Fig. 1:**
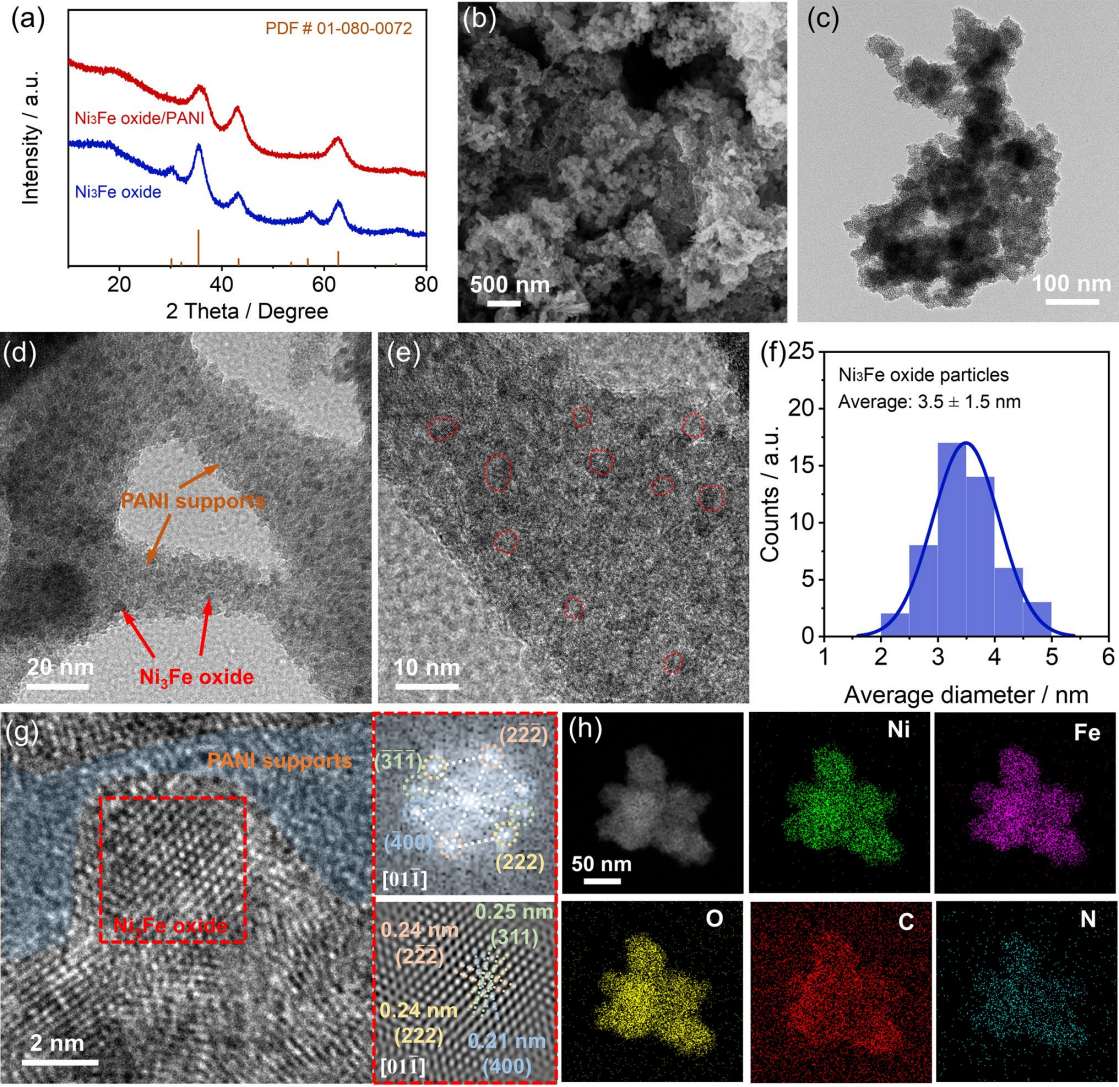
Crystal structure of Ni_3_Fe oxide/PANI catalyst. **a** XRD pattern, **b** SEM image, **c** TEM image, and **d, e** HRTEM images of Ni_3_Fe oxide/PANI catalysts. **f** Diameter distribution of Ni_3_Fe oxide on PANI supports. **g** HRTEM images of Ni_3_Fe oxide/PANI catalysts and corresponding FFT image of Ni_3_Fe oxide, and **h** corresponding element mapping of Ni_3_Fe oxide/PANI catalysts

In a typical synthesis of Ni_3_Fe oxide/PANI and other control samples, the commercial PANI, applied as the support to load catalysts, was first dispersed uniformly into solvent by ultrasonication, followed by a subsequent dispersion of metal ion and hexamethylenetetramine prior to further solvothermal reaction. The as-obtained precursor was calcined in air to obtain the final catalyst (details shown in Experimental Section). For comparison, Ni_3_Fe oxide, Ni oxide/PANI and Fe oxide/PANI were also prepared through the same process without adding PANI, Fe, and Ni sources, respectively. The crystal structure of obtained catalysts was first explored by X-ray diffraction (XRD) pattern in Fig. 1a and S1. Ni_3_Fe oxide/PANI and Ni_3_Fe oxide show main diffraction peaks located at 35.7°, 43.4°, and 62.9°, corresponding to the (311), (400), and (440) crystal plane, which can be indexed to the cubic Ni/Fe spinel oxide structure (PDF # 01-080-0072) [35]. More importantly, the particle size of Ni_3_Fe oxide in Ni_3_Fe oxide/PANI was calculated to be 2.5 nm according to the Scherrer equation [36]. In comparison, Fe oxide/PANI exhibits distinctive peaks corresponding to the iron oxide owing to the easy aggregation of iron oxides, while Ni oxide/PANI does not present a clear diffraction peak of nickel oxide given that the interaction between nickel oxide and PANI could suppress the aggregation of nickel oxide. Moreover, we can observe a broad diffraction peak at ~ 21° that is attributed to the characteristic peak of the PANI support due to the amorphous structure [37]. The microstructure of Ni_3_Fe oxide/PANI was further explored by scanning electron microscopy (SEM). As shown in Fig. 1b and S2, Ni_3_Fe oxide/PANI exhibits the structure of stacking nanoparticles without any independent large particles, which means that the Ni_3_Fe oxide is well dispersed on the PANI support.

We further explore the supported structure of Ni_3_Fe oxide/PANI by transmission electron microscopy (TEM). Selecting independent Ni_3_Fe oxide/PANI particles for microstructure analysis, as shown in Fig. 1c, Ni_3_Fe oxide/PANI exhibits a continuous porous structure and rough surface. The enlarged TEM images in Figs. 1d and S3 reveal that Ni_3_Fe oxides are uniformly supported on the PANI support without obvious accumulation. As shown in Fig. 1e, f, Ni_3_Fe oxide with an average diameter of 3.5 ± 1.5 nm is monodispersed on the PANI support, which is well consistent with the XRD results. We further analyze the interface structure between Ni_3_Fe oxide and PANI support through high-resolution TEM. An obvious hetero-interface between Ni_3_Fe oxide, with lattice fringes, and PANI support, with amorphous structures, is observed in Fig. 1g. We conduct a fast Fourier transform (FFT) to obtain the reciprocal space of Ni_3_Fe oxide. Four planes of (311), $$(2\,\overline{2}\,\overline{2})$$, (400), and (222) along the [$$$$0\, 1\, \overline{1}$$$$] zone axis, which can be indexed in the HRTEM image, further prove that Ni_3_Fe oxide exhibits the spinel structure [38, 39]. The high-angle annular dark-field scanning TEM (HAADF-STEM) image and corresponding EDX elemental mapping in Fig. 1h demonstrated that the Ni, Fe, O, C, and N elements are uniformly distributed in a monodispersed Ni_3_Fe oxide/PANI particle, further indicating the uniform dispersion of Ni_3_Fe oxide on the PANI support. As further confirmed via the thermogravimetric (TG) characterization in Fig. S4, the mass content of Ni_3_Fe oxide is around 74.12% in the Ni_3_Fe oxide/PANI composite, which is consistent with the amount of raw materials added during the synthesis. In addition, calcination of Ni_3_Fe oxide/PANI at 350 °C will not damage the skeletal structure of PANI supports (Fig. S5), whereas calcination to remove HCl dopants may enhance the interaction between Ni_3_Fe oxide and PANI skeleton [40].

### Catalyst–Support Interaction

**Fig. 2:**
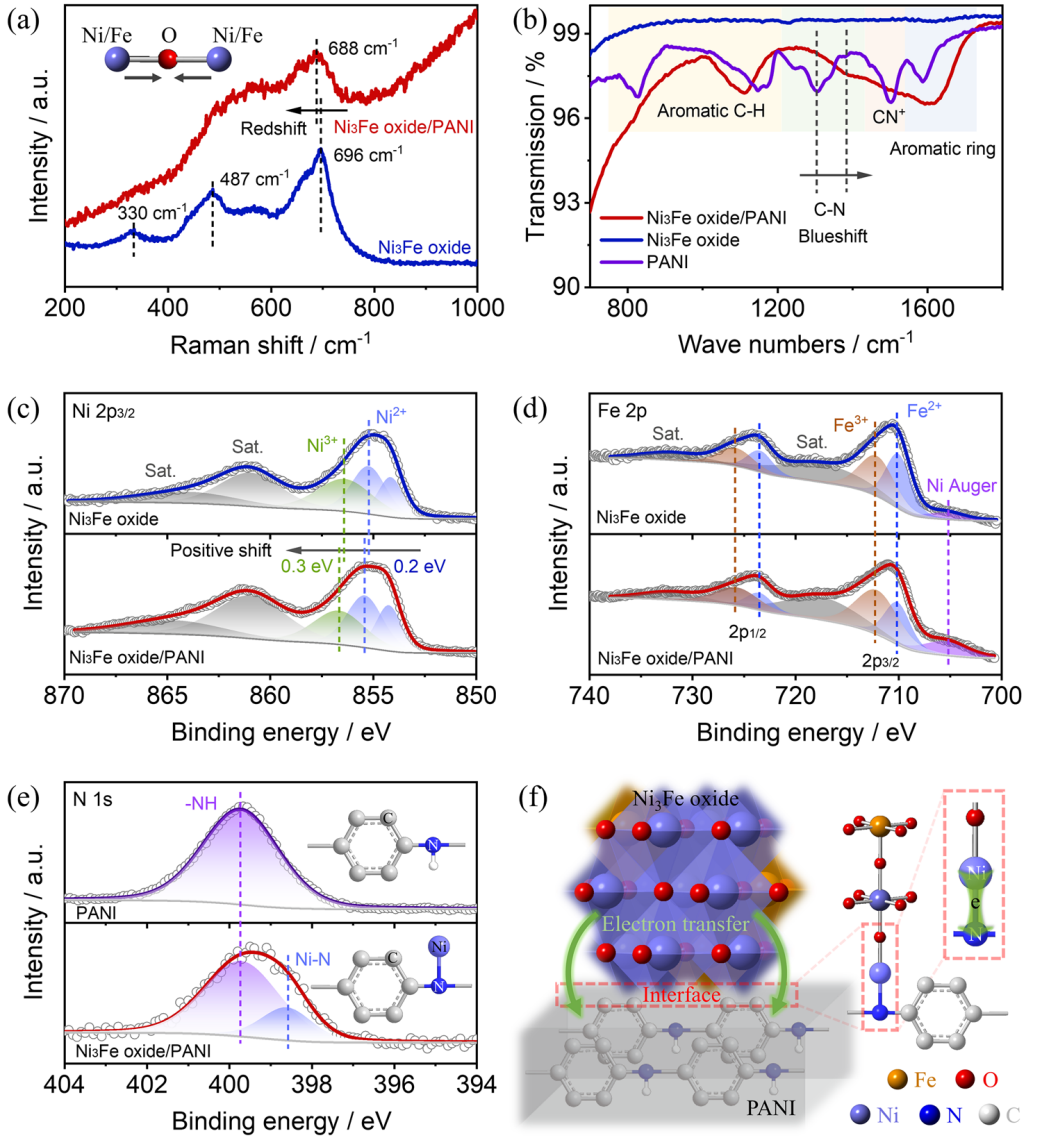
Catalyst–support interaction in Ni_3_Fe oxide/PANI catalyst. **a** Raman scattering spectra of Ni_3_Fe oxide and Ni_3_Fe oxide/PANI catalysts (insert: the stretching vibration illustration of metal-O bonds). **b** FTIR characterization of PANI, Ni_3_Fe oxide, and Ni_3_Fe oxide/PANI catalysts. XPS characterization: **c** Ni 2*p*_3/2_ for Ni_3_Fe oxide and Ni_3_Fe oxide/PANI catalysts, **d** Fe 2*p* for Ni_3_Fe oxide and Ni_3_Fe oxide/PANI catalysts, and **e** N 1*s* for PANI and Ni_3_Fe oxide/PANI catalysts. **f** Scheme illustration of the electron transfer at the interface of Ni_3_Fe oxides and PANI supports

The hetero-interface between the amorphous PANI support and crystallized Ni_3_Fe oxide would induce charge rearrangement to regulate the catalytic activities. We further explore the electronic structure of catalysts Ni_3_Fe oxide/PANI, Ni_3_Fe oxide, and PANI via Raman, Fourier transform infrared spectroscopy (FTIR), and X-ray photoelectron spectroscopy (XPS) to probe the catalyst–support interaction. As shown in Fig. 2a, the Ni_3_Fe oxide exhibited three obvious peaks at 330, 487, and 696 cm^−1^ that are ascribed, respectively, to the E_g_, T_2g_, and A_1g_ modes of the spinel oxide [41]. Importantly, Ni_3_Fe oxide/PANI shows a redshift of the A_1g_ mode compared to Ni_3_Fe oxide, indicating a shortened Ni/Fe–O bond after introducing PANI [42]. The shortened Ni/Fe–O bond can lead to an increase in the Ni–O covalency, which is beneficial for OER activities. Raman spectra of Fe oxide/PANI and Ni oxide/PANI are also presented in Fig. S6. Fe oxide/PANI exhibited obvious Fe_2_O_3_ peaks while Ni oxide/PANI delivered weak NiO peaks, indicating a strong binding force between the Ni oxide and PANI support to inhibit Ni oxide aggregation. PANI and other PANI-containing catalysts exhibited Raman peaks around 1580 and 1350 cm^−1^ that were associated with C=C stretching deformation of benzenoid rings and C–N^+^ vibration mode of the quinoid ring, respectively [43]. In addition, the changes in the electronic structure of PANI supports can also be explored through FTIR analysis. The strong absorption around 600 cm^−1^ is attributed to the Ni/Fe–O bond (Fig. S7), and peaks at 1500 and 1304 cm^−2^ are indexed, respectively, to C=C stretching mode and C–N^+^ vibration (Fig. 2b) [44]. Specifically, Ni_3_Fe oxide/PANI exhibited a blueshift of C–N^+^ bonds compared to PANI, indicating that the PANI support acquires electrons from the Ni_3_Fe oxide through N atoms.

The insight into electronic interaction between the Ni_3_Fe oxide and PANI support is further explored through the XPS. As shown in Fig. 2c, the Ni 2*p*_3/2_ spectra of both the Ni_3_Fe oxide and Ni_3_Fe oxide/PANI could be deconvoluted into two peaks attributed to Ni^2+^, one peak of Ni^3+^, and two satellite peaks due to the existent of two types of Ni coordination, Ni–O–Ni and Ni–O–Fe. More importantly, Ni_3_Fe oxide/PANI presented a positive shift of Ni^2+^ and Ni^3+^ peaks by about 0.2 and 0.3 eV compared to the Ni_3_Fe oxide catalyst, indicating that the valence state of Ni increases after introducing the PANI support, which is consistent with the Raman results [45]. Differently, Ni_3_Fe oxide and Ni_3_Fe oxide/PANI exhibited the same binding energy of Fe^2+^, Fe^3+^, and two satellite peaks (Fig. 2d), revealing that the PANI supports have a negligible influence on the electronic structure of Fe ion [46]. This conclusion can also be verified through the O 1*s* spectra in Fig. S8. The Ni-lattice O bond exhibited a 0.25 eV positive shift in binding energy, much higher than that of the Fe-lattice O bond (0.1 eV). In addition, Ni_3_Fe oxide/PANI exhibits a positive shift of binding energy for *OH and *H_2_O species compared to Ni_3_Fe oxide, revealing that PANI supports can regulate the adsorption energy of oxygen species on Ni_3_Fe oxide catalysts [47]. Considering the XPS results, we further analyze the N 1*s* spectra to probe the changes in the electronic structure of PANI supports. As shown in Fig. 2e, Ni_3_Fe oxide/PANI displays two peaks at 399.7 and 398.6 eV that are, respectively, ascribed to N–H and Ni–N bonds considering the different binding energy of Fe–N [48, 49] and Ni–N [50] bonds (Fig. S9), while PANI just exhibits a peak at 399.7 eV attributed to N–H bond [51]. Taken together, we conclude that the heterostructure between the Ni_3_Fe oxide and PANI support was formed via the strong interaction between the Ni atom in the Ni_3_Fe oxide and the N atom in the PANI support, in which electron transfers from the Ni_3_Fe oxide to the PANI support, as shown in Fig. 2f. Such type of charge rearrangement can induce high valence Ni, which is conducive to the OER activity under an alkaline electrolyte.

### Electrocatalytic Performance and Application in Zn-Air Batteries

To investigate the effect of catalyst–support interaction on electrochemical activities, we further evaluate the OER performance of Ni_3_Fe oxide/PANI and control catalysts through a typic three-electrode system based on a rotating disk electrode (RDE) instrument in O_2_-saturated 0.1 M KOH. The optimum ratio of Ni and Fe in PANI was first explored in Fig. S10. We found that Ni_3_Fe oxide/PANI delivers the best OER activity considering its low charge transfer impedance, overpotential, and Tafel slope. Besides, we also explored the effect of air calcination of catalysts on OER activity in Fig. S11 and found that the calcination process can improve OER activities and decrease charge transfer impedance of Ni_3_Fe oxide/PANI, Ni oxide/PANI, and Fe oxide/PANI, which means that calcination treatment can enhance the interfacial interaction between the Ni_3_Fe oxide and PANI support.

**Fig. 3:**
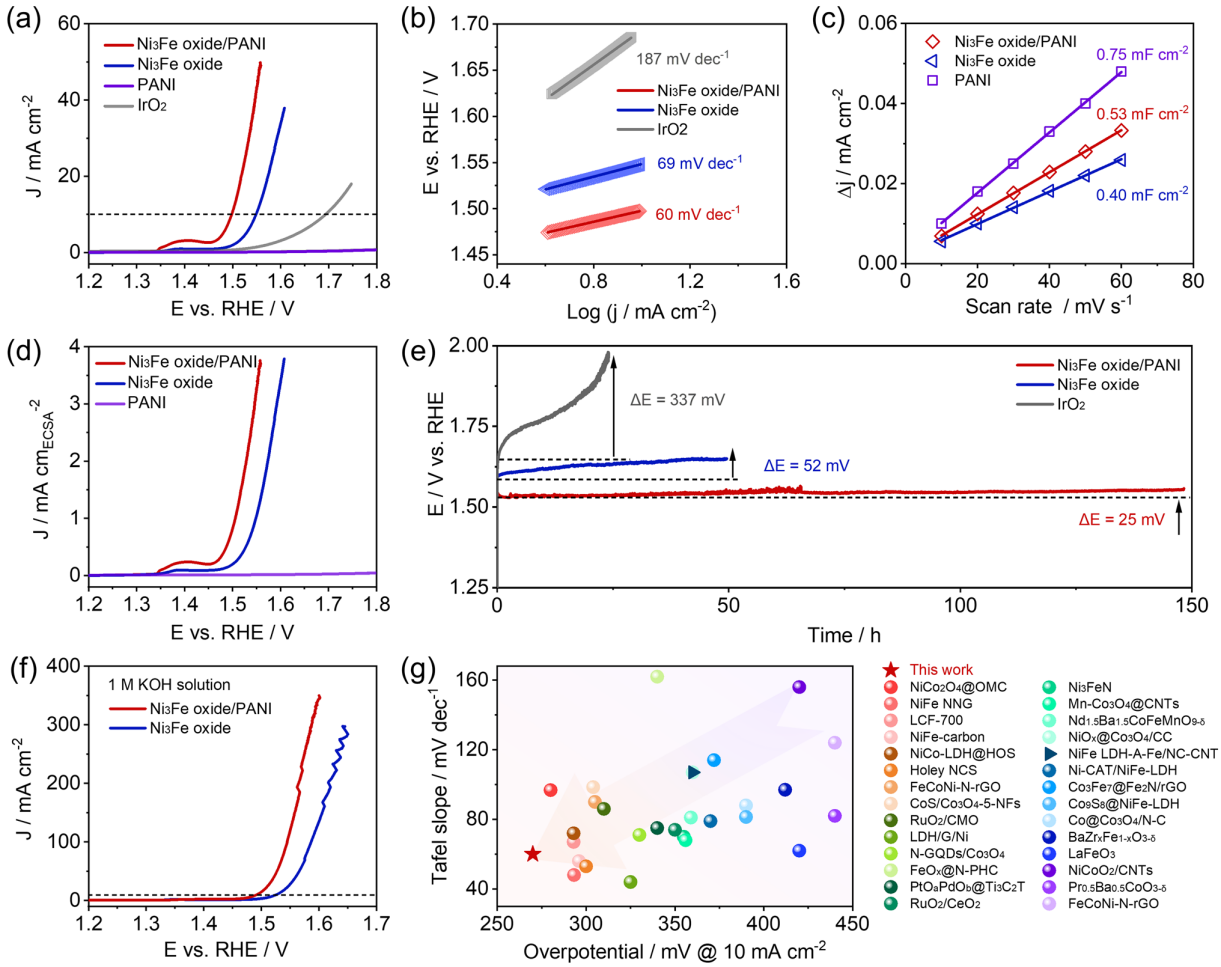
OER activity and stability in 0.1 M KOH. **a** OER polarization curves and **b** corresponding Tafel slopes for the Ni_3_Fe oxide, PANI, commercial IrO_2_, and Ni_3_Fe oxide/PANI catalysts. **c** Half of the difference in current density at 0.92 V (vs. RHE) versus scan rate from 10–60 mV s^−1^ for the Ni_3_Fe oxide, PANI, and Ni_3_Fe oxide/PANI catalysts. **d** Comparing the OER activity based on the ECSA value for Ni_3_Fe oxide, PANI, and Ni_3_Fe oxide/PANI catalysts. **e** OER stability at 10 mA cm^−2^ for Ni_3_Fe oxide, commercial IrO_2_, and Ni_3_Fe oxide/PANI catalysts. **f** OER polarization curves for Ni_3_Fe oxide and Ni_3_Fe oxide/PANI catalysts in 1 M KOH. **g** Comparison of the OER activity in alkaline solution of this work with reported catalysts

We then choose the optimum Ni_3_Fe oxide/PANI for further investigating the impact of the interaction between Ni_3_Fe oxide and PANI on catalytic activity. OER activities were first acquired from the linear sweep voltammetry (LSV) polarization curve in Fig. 3a. Ni_3_Fe oxide/PANI delivered a lower OER overpotential of 270 mV at 10 mA cm^−2^ than that of 320 mV for Ni_3_Fe oxide and 390 mV for commercial IrO_2_, while the PANI support showed negligible OER activities. Moreover, Ni_3_Fe oxide/PANI achieves a Tafel slope of 60 mV dec^−1^, which is lower than that of 69 mV dec^−1^ for Ni_3_Fe oxide and 187 mV dec^−1^ for IrO_2_ (Fig. 3b), further indicating enhanced OER kinetics after introducing PANI into the Ni_3_Fe oxide. The electrochemically active surface area (ECSA) is calculated through the double-layer capacitance (*C*_dl_) to evaluate the intrinsic activity (Fig. S12) [52]. The C_dl_ value of Ni_3_Fe oxide/PANI, Ni_3_Fe oxide, and PANI are calculated, respectively, to be 0.53, 0.40, and 0.75 mF cm^−2^ in Fig. 3c. The increased C_dl_ suggests that the PANI support can help increase the dispersion of the Ni_3_Fe oxide. Further, the intrinsic activity of Ni_3_Fe oxide/PANI, Ni_3_Fe oxide, and PANI was acquired based on the ECSA as shown in Fig. 3d. The intrinsic activity follows the sequence of Ni_3_Fe oxide/PANI > Ni_3_Fe oxide > PANI, strongly validating that the PANI support could significantly promote the catalytic activities of Ni_3_Fe oxide although PANI itself is almost inert toward the OER. Specifically, Ni_3_Fe oxide/PANI exhibits 3.84 times of intrinsic activity at an overpotential of 300 mV than Ni_3_Fe oxide.

In addition, we also measure the catalytic activity of Ni oxide/PANI and Fe oxide/PANI to highlight the origin of interaction between Ni_3_Fe oxide and PANI on OER activity. As shown in Figs. S13 and S14, Ni oxide/PANI requires a smaller overpotential of 330 mV at 10 mA cm^−2^ than that of 465 mV for Fe oxide/PANI and delivers a 17.6 times higher intrinsic activity at 1.62 V versus RHE than Fe oxide/PANI, indicating that the interaction between Ni oxide and PANI has a greater impact on OER activity than that between Fe oxide and PANI. Moreover, compared to Fe oxide/PANI and Ni oxide/PANI, Ni_3_Fe oxide/PANI exhibited even lower overpotential at 10 mA cm^−2^, smaller Tafel slopes (Fig. S15a), and higher specific/mass activity (Fig. S15b). The charge transfer impedance was obtained through electrochemical impedance spectroscopy (EIS) at 0.75 V versus Hg/HgO in Fig. S15c. Ni_3_Fe oxide/PANI showed a lower charge transfer impedance than Ni_3_Fe oxide, and Ni oxide/PANI delivered a significantly lower charge transfer impedance than Fe oxide/PANI, suggesting that the interfacial Ni–N bond between the Ni_3_Fe oxide and PANI support could improve the electron transfer rate during the catalytic process.

In addition to activity, stability is another indicator for evaluating the catalyst performance. We applied chronopotentiometry at 10 mA cm^−2^ to investigate the OER durability of Ni_3_Fe oxide/PANI, Ni_3_Fe oxide, and IrO_2_ in 0.1 M KOH. As illustrated in Fig. 3e, Ni_3_Fe oxide/PANI only experienced a slight increase in overpotential of 25 mV after 150 h, in comparison with that of 52 mV after 50 h for Ni_3_Fe oxide and 337 mV after 20 h for IrO_2_, indicating that the PANI support can stabilize Ni_3_Fe oxide via the Ni–N interfacial bond. To pursue high current density, we also evaluated the OER catalytic activity in 1 M KOH in Fig. 3f. Ni_3_Fe oxide/PANI and Ni_3_Fe oxide catalysts exhibited the potential of 1.58 and 1.65 V at 300 mA cm^−2^, indicating the viability of Ni_3_Fe oxide/PANI in Zn-air batteries to operate at high current densities. To highlight the superiority of our work, we further compare the designed Ni_3_Fe oxide/PANI catalyst with the relevant catalysts reported in recent years in Fig. 3g. Notably, Ni_3_Fe oxide/PANI shows a lower overpotential and smaller Tafel slope compared to reported catalysts in an alkaline medium, highlighting the advantage of our work. Detailed information about reported catalysts is listed in Table S1.

**Fig. 4:**
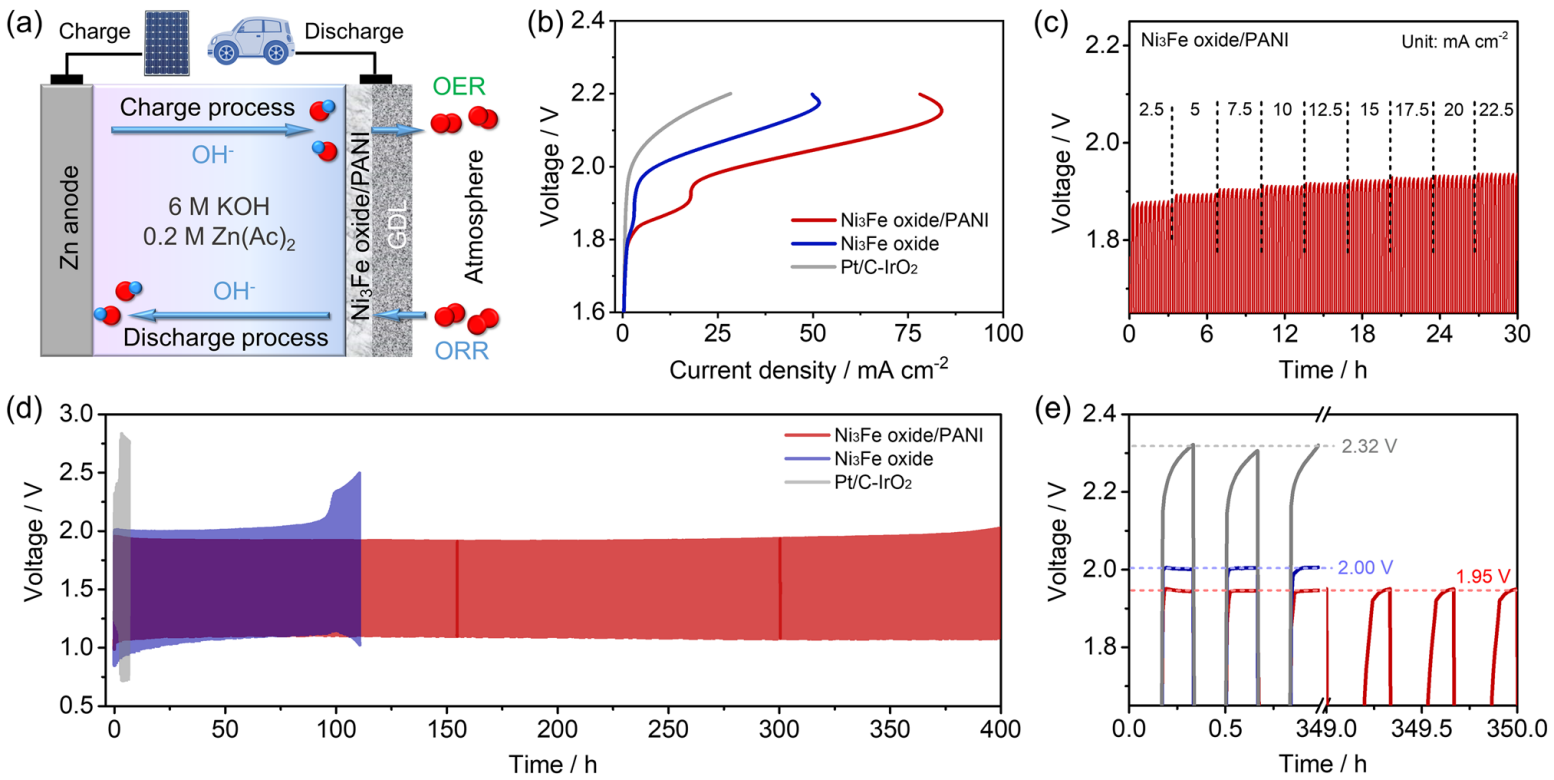
Performance of rechargeable Zn-air batteries. **a** Schematically illustration of the primary Zn-air battery. **b** Charge polarization curves of Zn-air batteries with Ni_3_Fe oxide, Ni_3_Fe oxide/PANI and Pt/C-IrO_2_ as cathodic catalysts. **c** Galvanostatic charge test at the current density from 2.5 to 22.5 mA cm^−2^ of Zn-air batteries with Ni_3_Fe oxide/PANI. **d, e** Galvanostatic cycling tests of Zn-air batteries at 10 mA cm^−2^ (20 min per cycle) with Ni_3_Fe oxide, Ni_3_Fe oxide/PANI and Pt/C-IrO_2_ as cathodic catalysts

The excellent activity and stability of Ni_3_Fe oxide/PANI indicate its potential for application in practical devices. We further consider using Ni_3_Fe oxide/PANI as an oxygen catalyst in rechargeable Zn-air batteries. As shown in Fig. 4a, the air cathode was fabricated with Ni_3_Fe oxide/PANI or Ni_3_Fe oxide or Pt/C-IrO_2_ catalysts coated on the gas diffusion layer, while using the fresh Zn plate and 6 M KOH + 0.2 M Zn(Ac)_2_ solution as the anode and electrolyte, respectively. As shown in Fig. S16a, although both Ni_3_Fe oxide/PANI and Ni_3_Fe oxide exhibit poor ORR activity, Ni_3_Fe oxide/PANI achieves a potential 31 mV higher than Ni_3_Fe oxide at 2 mA cm^−2^, which is mainly due to the contribution of the PANI support. After being applied in Zn-air batteries, Ni_3_Fe oxide/PANI delivers a higher power density of 135 than 92 mW cm^−2^ for Ni_3_Fe oxide (Fig. S16b) only slightly lower than that of 180 mW cm^−2^ for Pt/C-IrO_2_ (Fig. S16c). Therefore, Ni_3_Fe oxide/PANI can be applied as oxygen catalysts in rechargeable Zn-air batteries.

The charge polarization curve was further evaluated considering the excellent OER catalytic activity of designed catalysts. As shown in Fig. 4b, Ni_3_Fe oxide/PANI-assembled Zn-air battery exhibits lower charge potential than Ni_3_Fe oxide and Pt/C-IrO_2_ within 0–80 mA cm^−2^, which is in good agreement with the tendency of OER activities. Specifically, Ni_3_Fe oxide/PANI exhibits a charge potential 0.11 V lower than that of Ni_3_Fe oxide at 50 mA cm^−2^. We further investigate the rate performance of Ni_3_Fe oxide/PANI at different current densities from 2.5 to 22.5 mA cm^−2^ in Fig. 4c (tested for 10 cycles at each current density and 20 min per cycle). In detail, Ni_3_Fe oxide/PANI delivered the charge voltage of 1.877, 1.892, 1.905, 1.911, 1.918, 1.922, 1.928, 1.931, and 1.936 V at 2.5, 5, 7.5, 10, 12.5, 15, 17.5, 20, and 22.5 mA cm^−2^ without significant voltage attenuation under each current density. As reported, the high charging voltage caused by the sluggish kinetic of OER will heavily affect the performance of fast-charging Zn-air battery [53–59]. Therefore, the designed catalyst exhibited a reduced charging voltage even at high current density and repeated testing (Fig. S17), finally demonstrating its potential in fast charging. Furthermore, the charge/discharge cycling stability of Zn-air batteries assembled with Ni_3_Fe oxide/PANI, Ni_3_Fe oxide, and Pt/C-IrO_2_ was evaluated through galvanostatic tests at 10 mA cm^−2^. As shown in Fig. 4d, Ni_3_Fe oxide/PANI-assembled Zn-air battery achieves a stable cycling performance for over 400 h, while Ni_3_Fe oxide and Pt/C-IrO_2_ catalysts showed significant voltage increases after cycling for only 100 and 10 h, respectively. Specifically, Ni_3_Fe oxide/PANI, Ni_3_Fe oxide, and Pt/C-IrO_2_ catalysts-assembled Zn-air batteries delivered initial charge voltage of 1.95, 2.00, and 2.32 V, respectively, while Ni_3_Fe oxide/PANI maintained a voltage of 1.95 V after cycling for 350 h (Fig. 4e), implying the excellent cycling stability of the designed Ni_3_Fe oxide/PANI catalyst, which outperforms the Ni_3_Fe oxide and Pt/C-IrO_2_ counterparts and most other reported transition metal-based catalysts as listed in Table S2.

### Catalytic Mechanism Exploration

**Fig. 5:**
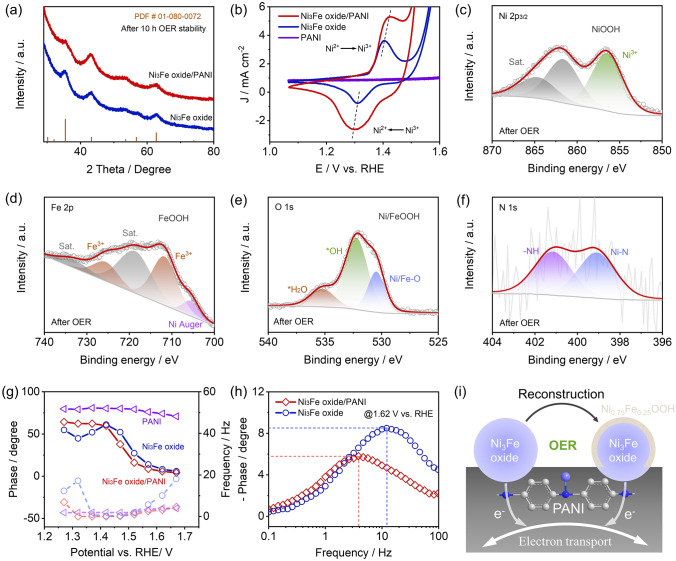
Electrocatalytic mechanism of Ni_3_Fe oxide/PANI catalyst. **a** XRD patterns for Ni_3_Fe oxide, and Ni_3_Fe oxide/PANI catalysts after 10 h OER stability test at 10 mA cm^−2^. **b** CV curves for Ni_3_Fe oxide, PANI, and Ni_3_Fe oxide/PANI catalysts under the potential range of 1.0–1.6 V versus RHE. XPS patterns for Ni_3_Fe oxide/PANI catalysts after 10 h OER stability test at 10 mA cm^−2^: **c** Ni 2*p*_3/2_, **d** Fe 2*p*, **e** O 1*s* and **f** N 1*s*. **g** Comparison of phase value and Frequency of Ni_3_Fe oxide, PANI, and Ni_3_Fe oxide/PANI catalysts within 1.27–1.67 V versus RHE. **h** EIS bode plots of Ni_3_Fe oxide and Ni_3_Fe oxide/PANI catalysts at 1.62 V versus RHE. **i** Scheme illustration of the Ni_3_Fe oxide/PANI catalyst during OER process

Ni_3_Fe oxide/PANI may undergo reconstruction during the OER process; therefore, further exploration of actual catalytic active sites is needed. To gain deep insight into the actual catalytic site, we explore the structural information of Ni_3_Fe oxide/PANI catalyst after 10 h OER test at 10 mA cm^−2^. As exhibited in Fig. 5a, Ni_3_Fe oxide/PANI and Ni_3_Fe oxide catalysts maintain the original spinel structure (PDF# 01-080-0072) after the stability test, indicating that the crystal structure of bulk Ni_3_Fe oxide is well maintained under highly oxidative conditions of the OER. Fe oxide/PANI and Ni oxide/PANI catalysts also maintained the original crystal structures after 100 cyclic voltammetry (CV) tests (Fig. S18a). Besides, we also studied the microstructure of Ni_3_Fe oxide/PANI after 10 h OER tests. As shown in Fig. S19a, b, the supported structure of Ni_3_Fe oxide/PANI catalyst remains unchanged after OER, while Ni_3_Fe oxides with particle sizes lower than 5 nm manifested a uniform dispersion on the PANI support. In particular, the hetero-interface between the Ni_3_Fe oxide and amorphous PANI support can be observed (Fig. S19c), and the (311) and (400) crystal planes of Ni_3_Fe oxide were also found (Fig. S19d), further confirming that the crystal structure of Ni_3_Fe oxide/PANI is well maintained. However, transition metal compounds are prone to surface reconstruction during OER process, resulting in actual active sites to differ from fresh catalysts. Ni and Fe-based catalysts would present the reversible electrochemical redox reaction processes of Ni^2+^/Ni^3+^ and Fe^2+^/Fe^3+^ redox couples, which can be found from CV curves of Ni oxide/PANI and Fe oxide/PANI in Fig. S18b. Similarly, Ni_3_Fe oxide/PANI and Ni_3_Fe oxide demonstrated a redox couple of Ni^2+^/Ni^3+^ in Fig. 5b, indicating that Ni atoms serve as the main catalytic active site [25].

Considering that CV results indeed indicate the electrochemical reconstruction of Ni_3_Fe oxide/PANI, we further investigated the surface electronic structure through XPS. After 10 h OER tests, Ni 2*p*_3/2_ spectra only display a single peak of Ni^3+^, which is attributed to NiOOH, and two corresponding satellite peaks in Fig. 5c, while Fe 2*p* spectra can be deconvoluted into a doublet corresponding to Fe^3+^, which is indexed to FeOOH, and two satellite peaks in Fig. 5d [24, 39]. Moreover, the high-resolution O 1*s* spectra of Ni_3_Fe oxide/PANI after OER are also analyzed in Fig. 5e. O 1*s* spectra of Ni_3_Fe oxide/PANI can be deconvoluted into three peaks attributed to OER-involved oxygen species of ^*^OH and ^*^H_2_O, and a single metal–oxygen bond. Compared with the original Ni_3_Fe oxide/PANI with two Ni-lattice O and Fe-lattice O bonds, Ni_3_Fe oxide/PANI after OER just exhibits a single Ni/Fe-lattice O bond and a significantly increased *OH peak. Combining these XPS results, we speculate that Ni_3_Fe oxide in Ni_3_Fe oxide/PANI was converted into Ni_0.75_Fe_0.25_OOH after OER. Of note, the Ni–N bond still existed in the post-OER Ni_0.75_Fe_0.25_OOH/PANI catalyst, as seen from the N 1*s* spectra in Fig. 5f, indicating that the interface between the Ni_3_Fe oxide and PANI support was still maintained after OER. Moreover, the E_g_, T_2g_, and A_1g_ modes can also be found from Raman spectra of Ni_3_Fe oxide/PANI and Ni_3_Fe oxide catalysts after the stability test, while the redshift of A_1g_ mode of Ni_3_Fe oxide/PANI compared to Ni_3_Fe oxide can also be observed after OER as shown in Fig. S20, strongly confirming the superior electrochemical stability of Ni_3_Fe oxide/PANI. The interfacial Ni–N bond and redshift of A_1g_ mode further indicate that the surface reconstruction of Ni_3_Fe oxide does not affect the electron interaction with the PANI support.

After understanding the evolution of Ni_3_Fe oxide/PANI catalysts, we further explore the reaction mechanism through assessing pH dependence. As shown in Fig. S21, the OER activity of Ni_3_Fe oxide/PANI and Ni_3_Fe oxide catalysts was remarkably enhanced as the pH was raised from 12 to 14, in which the direct relationship between the OER activity and pH indicates that both Ni_3_Fe oxide/PANI and Ni_3_Fe oxide follow the lattice oxygen-mediated (LOM) pathway for the OER [60]. In particular, Ni_3_Fe oxide/PANI presented a reaction order of 0.305, surpassing that of 0.254 for Ni_3_Fe oxide, which is mainly due to the high covalent Ni–O bond induced by the PANI support. To further explore the electrocatalytic reaction kinetics and mass transfer behavior of designed catalysts, we conducted the operando EIS collected from 0.3 to 0.8 V versus Hg/HgO under a three-electrode system [61, 62]. As exhibited in Fig. S22, the charge transfer impedance gradually decreased while the applied potential increased for the Ni_3_Fe oxide, Ni_3_Fe oxide/PANI, Ni oxide/PANI, and Fe oxide/PANI; however, the PANI support displayed extremely high charge transfer impedance, indicating that the PANI support exhibited no OER activity. To gain mass transport and charge transfer rates, the corresponding bode plot was acquired from EIS results in Fig. S23. For comparison, the phase angle of the designed catalyst follows the sequence of Ni_3_Fe oxide/PANI < Ni_3_Fe oxide < Ni oxide/PANI < Fe oxide/PANI < PANI when the potential is below 1.62 V versus RHE. A low phase angle means that more electrons would participate in the OER process. When the potential is above 1.62 V versus RHE, Ni oxide/PANI exhibits a slightly lower phase angle than Ni_3_Fe oxide, which is mainly attributed to the enhanced charge transfer rate through the PANI support. The phase angle and frequency of Ni_3_Fe oxide/PANI, Ni_3_Fe oxide, and PANI within 0.4 and 0.8 V are further collected in Fig. 5g. The PANI support exhibited no electrochemical activity considering the high phase angle within the given potential range. In addition, Ni_3_Fe oxide/PANI delivered a lower phase angle and peak frequency than Ni_3_Fe oxide within the reaction ranges (> 1.42 V versus RHE), revealing a faster mass and electron transfer rate after incorporating the PANI support into Ni_3_Fe oxide. Specifically, the typical bode plot at 1.62 V versus RHE is demonstrated in Fig. 5h. The phase angle and frequency of Ni_3_Fe oxide/PANI are 5.9° and 3.9 Hz, respectively, which are significantly lower than 8.5° and 10.3 Hz for Ni_3_Fe oxide. Therefore, we believe that PANI support could promote the mass transfer and charge transfer rate of Ni_3_Fe oxide by increasing the Ni–O covalency and electron transfer at interfacial Ni–N bonds. Based on the above analysis, the reaction mechanism of the designed Ni_3_Fe oxide/PANI is illustrated in Fig. 5i. During OER, the surface of Ni_3_Fe oxide is electrochemically converted into Ni_0.75_Fe_0.25_OOH layer, and Ni–N bonds are formed at the hetero-interface of the Ni_3_Fe oxide and PANI support to increase the valence state of Ni atom and electron transfer rate to conductive PANI support, thus facilitating the electron and charge transfer rate in Ni_3_Fe oxide/PANI.

## Conclusions

In conclusion, we designed a composite catalyst with Ni_3_Fe oxide uniformly supported on PANI support, in which the particle size of Ni_3_Fe oxide was just 3.5 ± 1.5 nm. The hetero-interface between the crystalline Ni_3_Fe oxide and amorphous PANI support is well constructed, which can increase the Ni–O covalency in Ni_3_Fe oxide via the interfacial Ni–N bond, thus promoting the charge and mass transfer rate. The optimum Ni_3_Fe oxide/PANI delivers an ultra-low overpotential of 270 mV at 10 mA cm^−2^, small Tafel slope of 60 mV dec^−1^, and prolonged OER stability of 150 h at 10 mA cm^−2^. In addition, Ni_3_Fe oxide/PANI-assembled Zn-air batteries achieve an ultra-long cycle life of over 400 h at 10 mA cm^−2^ with a charge voltage lower than 2.0 V and an excellent rate performance, outperforming the Ni_3_Fe oxide and commercial Pt/C-IrO_2_ catalysts. This work provides new research insight via accurately matching conductive supports in designing supported catalysts for practical energy conversion devices.

## Supplementary Information

Below is the link to the electronic supplementary material.

Supplementary file1 (DOCX 6540 kb)
