# Supplementary material for: Catalyst–Support Interaction in Polyaniline-Supported Ni3Fe Oxide to Boost Oxygen Evolution Activities for Rechargeable Zn-Air Batteries
Source: Nanomicro Lett. 2024 Sep 21;17:6. doi: 10.1007/s40820-024-01511-4 (PMC11415562; doi:10.1007/s40820-024-01511-4)
Supplement: Supplementary file 1 — Supplementary file1 (DOCX 6540 kb) [file 40820_2024_1511_MOESM1_ESM.docx]

Supporting Information for

**Catalyst-Support Interaction in Polyaniline-Supported Ni_3_Fe Oxide to Boost Oxygen Evolution Activities for Rechargeable Zn-Air Batteries**

Xiaohong Zou ^1^, Qian Lu ^2, 3^, Mingcong Tang ^1^, Jie Wu ^1^, Kouer Zhang ^1^, Wenzhi Li ^1^, Yunxia Hu ^1^, Xiaomin Xu ^4^, Xiao Zhang ^1, 5, 6^*, Zongping Shao ^4,^ *, Liang An ^1, 5, 6,^ *

^1^Department of Mechanical Engineering, The Hong Kong Polytechnic University, Hung Hom, Kowloon, Hong Kong SAR, P. R. China

^2^Jiangsu Collaborative Innovation Center of Atmospheric Environment and Equipment Technology, Jiangsu Key Laboratory of Atmospheric Environment Monitoring and Pollution Control, School of Environmental Science and Technology, Nanjing University of Information Science and Technology, Nanjing 210044, P. R. China

^3^Department of Chemistry, The Chinese University of Hong Kong, Ma Lin building, Shatin 999077, Hong Kong SAR, P. R. China

^4^WA School of Mines: Minerals, Energy and Chemical Engineering (WASM-MECE), Curtin University, Perth, WA 6102, Australia

^5^Research Institute for Advanced Manufacturing, The Hong Kong Polytechnic University, Hung Hom, Kowloon, Hong Kong SAR, P. R. China

^6^Research Institute for Smart Energy, The Hong Kong Polytechnic University, Hung Hom, Kowloon, Hong Kong SAR, P. R. China

*Corresponding authors. E-mail: [liang.an@polyu.edu.hk](mailto:liang.an@polyu.edu.hk) (Liang An); [xiao1.zhang@polyu.edu.hk](mailto:xiao1.zhang@polyu.edu.hk) (Xiao Zhang); [shaozp@njtech.edu.cn](mailto:shaozp@njtech.edu.cn) (Zongping Shao)

S1 Experimental Section

**S1.1 Material characterization**

Scanning electron microscopy (SEM, TESCAN MIRA LMS) was taken to observe the morphology and nanoparticle dispersion of the obtained catalysts. X-ray diffraction (XRD) patterns were obtained by Rigaku SmartLab 9kW - Advance with Cu Kα radiation to detect the crystal structures of obtained catalysts. High-resolution transmission electron microscopy (HRTEM, JEOL JEM F200) was applied to explore the morphology and microstructure of catalysts with the corresponding element EDX mapping. Raman spectra were collected by RENISHAW Raman microscope with an excitation laser of 532 nm. Fourier transform infrared spectroscopy (FT-IR) test was taken via Vertex 70 FTIR spectrometer to detect the molecular structure and chemical composition of the obtained catalysts. X-ray photoelectron spectroscopy (XPS) was probed by Thermo Scientific ESCALAB Xi+ with Al Kα X-ray source and all the data were corrected with C 1s line at 284.6 eV. TG (thermogravimetric analysis) data were collected via NETZSCH TG 209F3 TGA209F3A-0171-L with the temperature from 30 to 1000 ℃.

**S1.2 Electrochemical tests**

The OER performance of these catalysts was recorded at potentiostation (Biologic instrument) with a rotation speed controller (Keruite Analytical Instrument Co., Ltd) by a standard three-electrode electrolytic cell with the alkaline solution, in which the obtained catalyst coated at the glassy carbon electrode served as the working electrode, the graphene rod acted as the counter electrode, and Hg/HgO in 1 M KOH solution was used as the reference electrode. Before preparing the working electrode, a uniform slurry needs to be made. In detail, 5 mg catalysts and 2.5 mg acetylene black were added into 0.5 mL mixed solution (isopropanol and Nafion with a volume ratio of 9:1) with vigorous ultrasound treatment. Subsequently, 5 μL of the prepared ink was drop-cast onto a glassy carbon electrode (GCE) with a catalyst loading of 0.2~0.3 mg cm^−2^. The electrochemistry performances were recorded in an O_2_-saturated 0.1 M, 1 M, and 0.01 M KOH aqueous electrolyte with a rotation speed of 1600 rpm for the working electrode. In detail, the OER performance was tested by linear sweep voltammetry (LSV) with *iR*-compensation at a scan rate of 5 mV s^−1^ and cyclic voltammetry (CV) at a scan rate of 20 mV s^−1^ within 0.2 to 1.0 V versus Hg/HgO, respectively [S1]. The above potentials were corrected to the RHE scale via a calibration equation given below:

$$E_{RHE}= E_{Hg/HgO}+0.059pH+0.098$$

For the durability tests, catalysts of Ni_3_Fe oxide/PANI, Ni_3_Fe oxide, and IrO_2_ were coated on the carbon paper with a mass loading around 1 mg cm^-2^ and chronopotentiometry was conducted at a constant current density of 10 mA cm^−2^ for the OER in the 0.1 M KOH solution. The electrochemical impedance spectroscopy (EIS) measurements were obtained under a frequency range from 100000 to 0.1 Hz with various potentials 1.17 – 1.67 V vs. RHE. The electrochemically active surface area (ECSA) of catalysts was recorded through CV tests within 0.87-0.97 V vs. RHE under different scan rates of 10, 20, 30, 40, 50, and 60 mV s^-1^. The electrochemical double-layer capacitance (C_dl_) is calculated through the equation of$\text{C}_{\text{dl}}\text{=}\text{i}_{\text{c}}\text{/}\text{v}$, in which *i_c_* and *v* means half of the difference in current density at 0.92 V (vs. RHE) and scan rate, respectively. [2, 3] The ECSA is calculated through the equation of ECSA = C_dl_/C_s_, in which C_s_ = 40 μF cm^-2^. The intrinsic activity of designed catalyst can be calculated by J_ECSA_ = J / ECSA, in which the J means the current (mA) in OER LSV curve.

**S1.3 Fabrication and evaluation of Zn-air batteries**

Home-made Zn-air battery was assembled by an air cathode with catalysts loaded on a gas diffusion layer, a fresh metal anode (high purity Zn foil), and an electrolyte (6 M KOH + 0.2 M ZnAc_2_ aqueous solution). Typically, to prepare the air cathode for the Zn-air battery, the Ni_3_Fe oxide/PANI catalyst slurry prepared in the above electrochemical tests section was drop-cast on the surface of a gas diffusion layer with the catalyst mass loading of ~1 mg cm^-2^. For comparison, a mixture of Ni_3_Fe oxide, Pt/C and IrO_2_ with a weight ratio of 1:1 was also prepared using the same procedure as Ni_3_Fe oxide/PANI. All battery tests were recorded in the ambient atmosphere and at room temperature. The discharge/charge polarization curves (voltage-current density) were measured through the CHI 760E with a voltage step of 5 mV s^−1^. The galvanostatic charge/discharge curves at the current density of 10 mA cm^-2^ and charge/discharge rate test (at the current density 2.5-22.5 mA cm^-2^) were collected by the Neware equipment testing system (5 V, 20 mA or 5 V, 10 A).

**S2 Supplementary Figures and Tables**


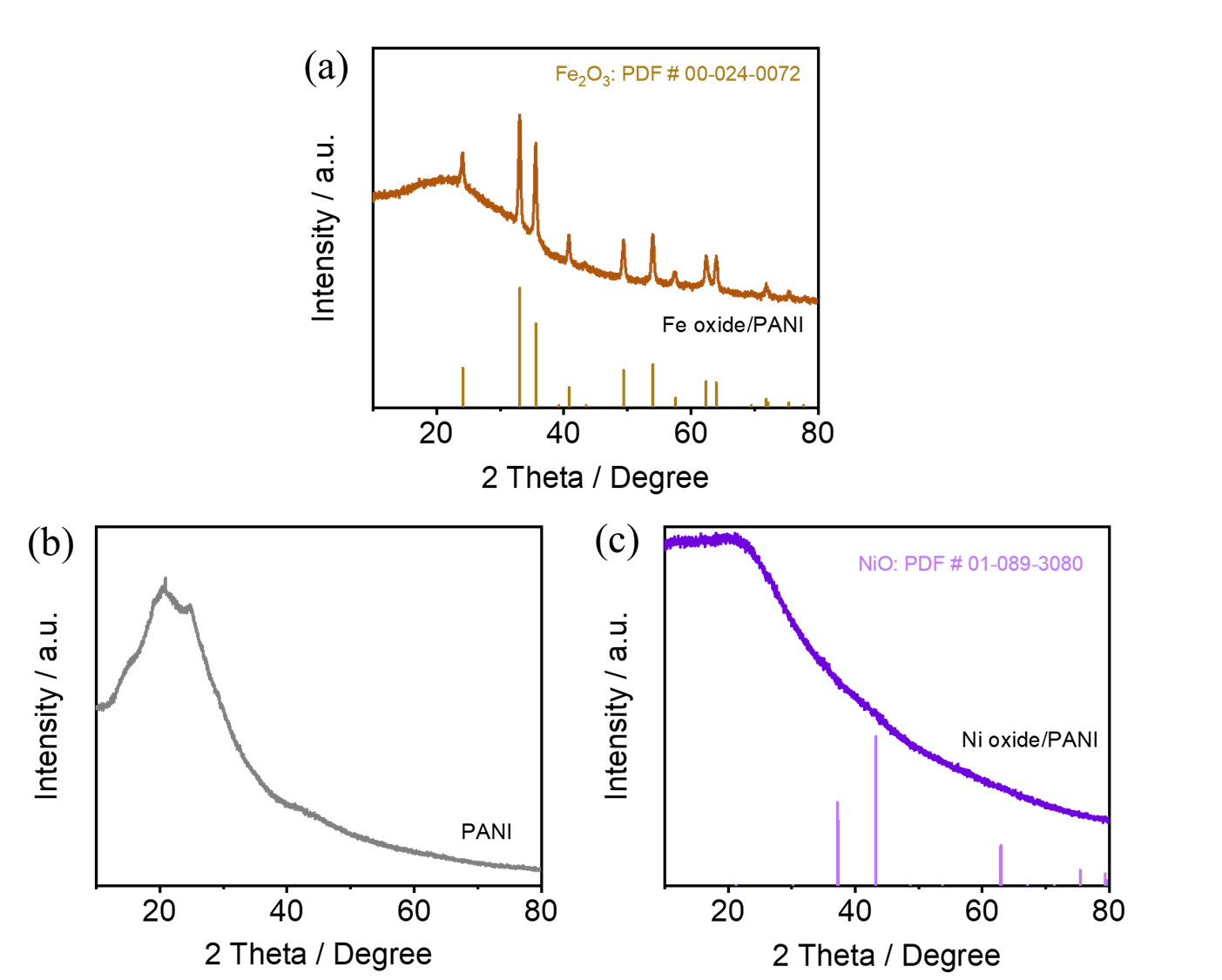


**Fig. S1** XRD patterns for the (**a**) Fe oxide/PANI, (**b**) PANI, and (**c**) Ni oxide/PANI


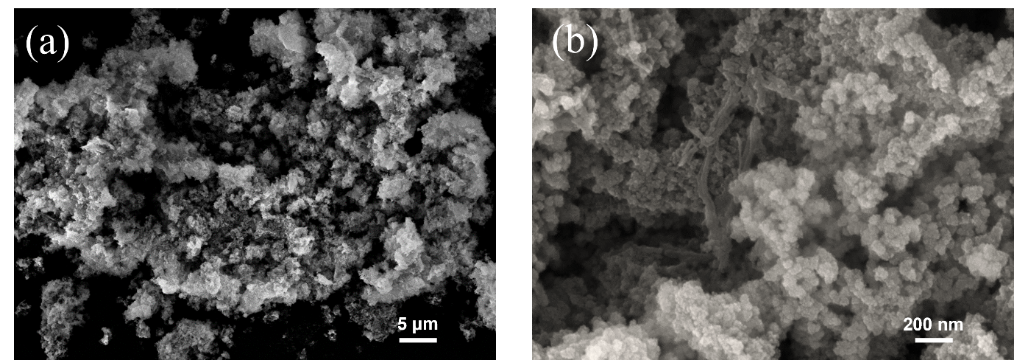


**Fig. S2** SEM images of Ni_3_Fe oxide/PANI catalysts


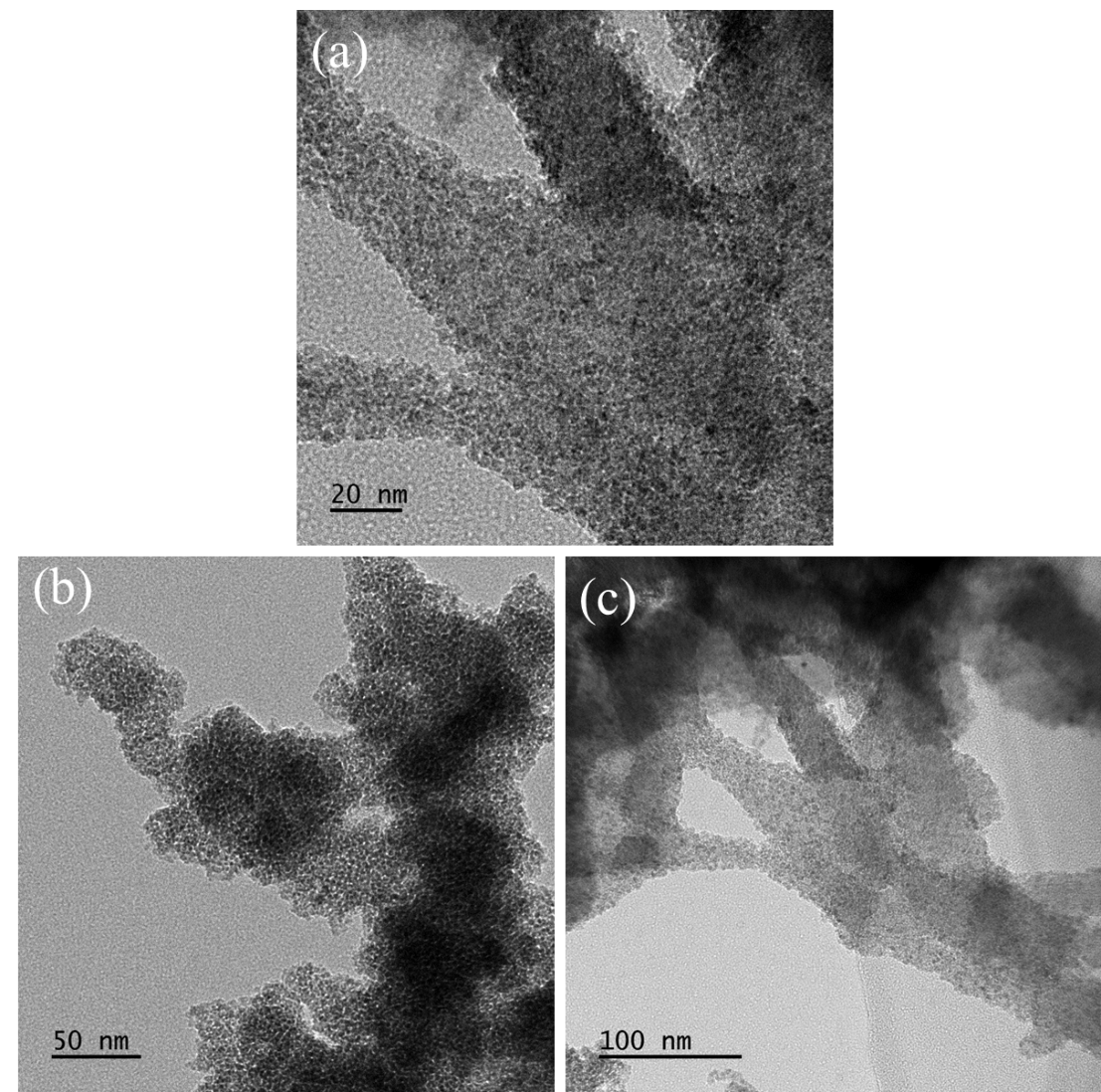


**Fig. S3** TEM images of Ni_3_Fe oxide/PANI catalysts


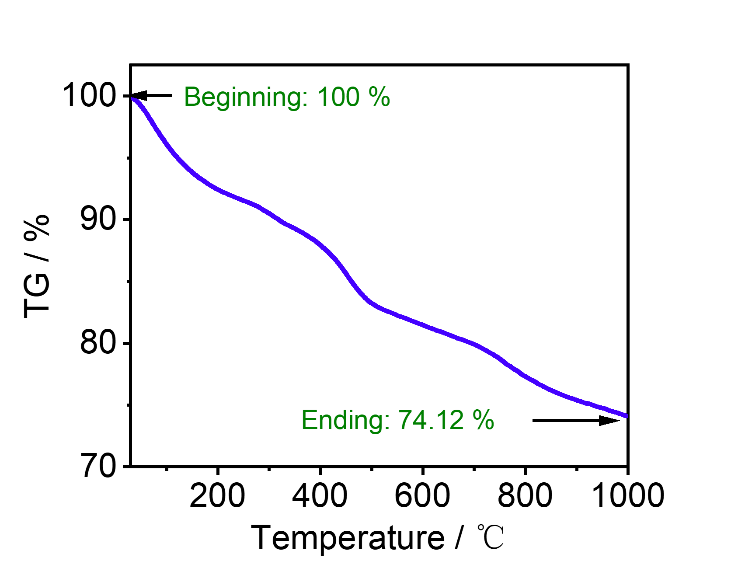


**Fig. S4** TG curve of Ni_3_Fe oxide/PANI catalysts


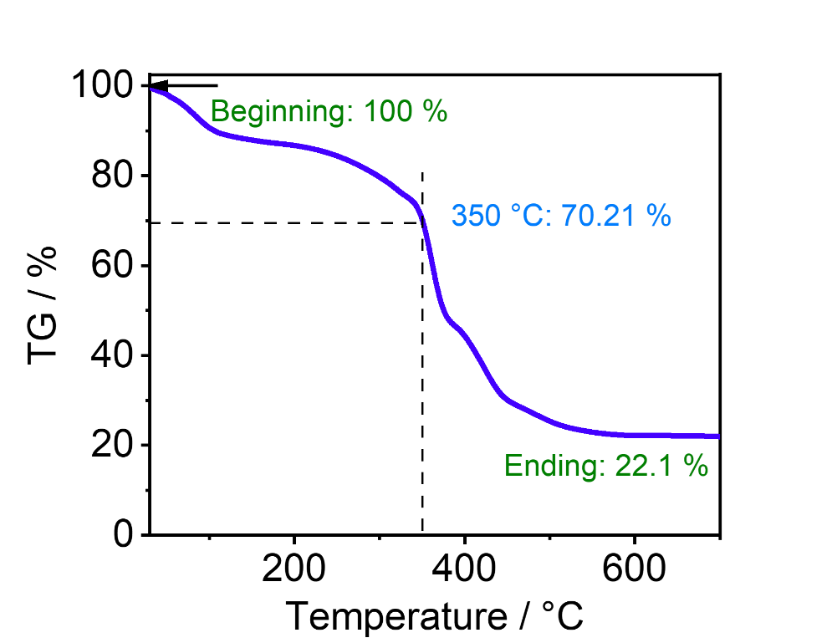


**Fig. S5** TG curve of Ni_3_Fe oxide/PANI without air calcination. The mass loss at 60 and 340 °C is attributed to the expulsion of HCl dopant from PANI and degradation of the PANI chain, respectively. The mass loss at 200 °C is attributed to the expulsion of adsorbed oxygen species from Ni_3_Fe oxide


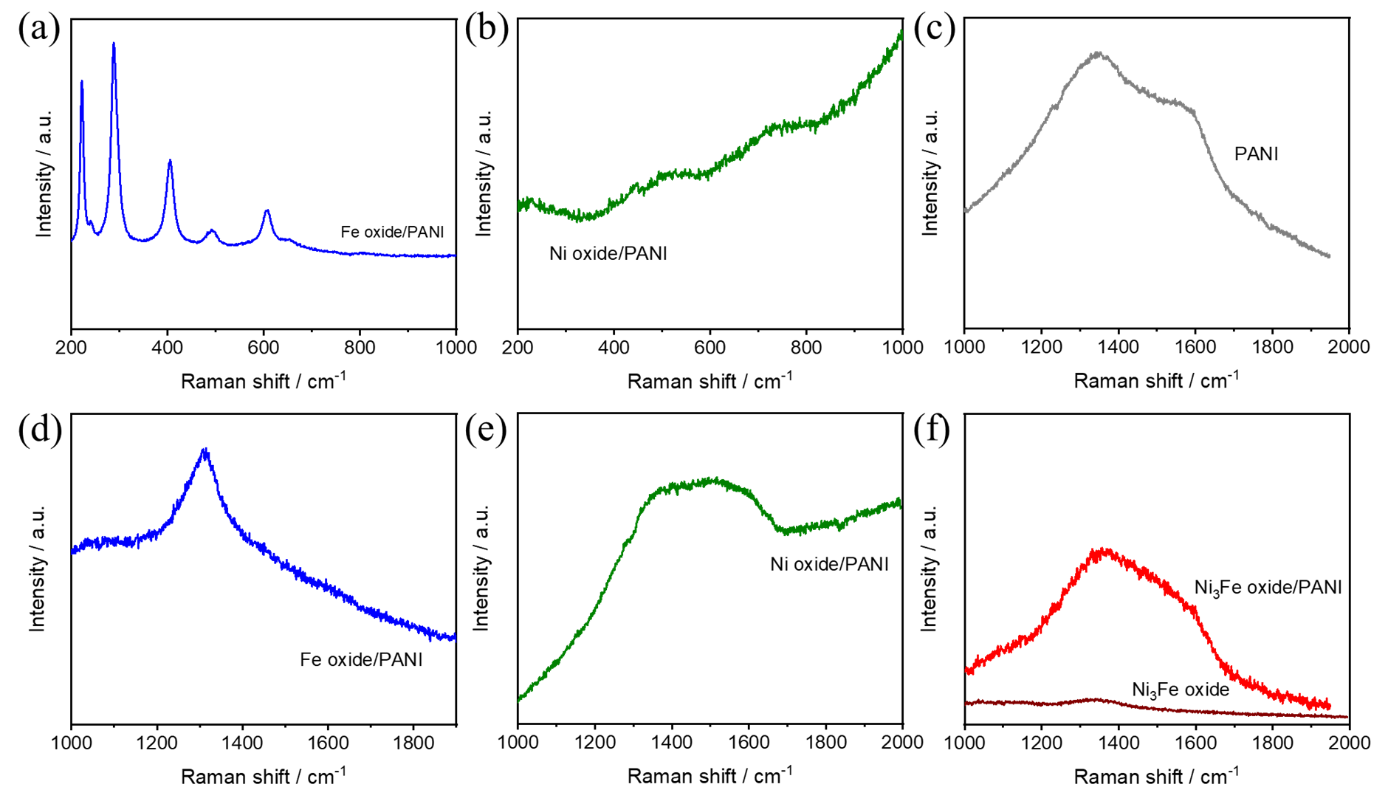


**Fig. S6** Raman patterns for (**a**) Fe oxide/PANI and (**b**) Ni oxide/PANI at the range of 200-1000 cm^-1^. Raman patterns for (**c**) PANI, (**d**) Fe oxide/PANI, (**e**) Ni oxide/PANI, (**f**) Ni_3_Fe oxide/PANI and Ni_3_Fe oxide at the range of 1000-2000 cm^-1^


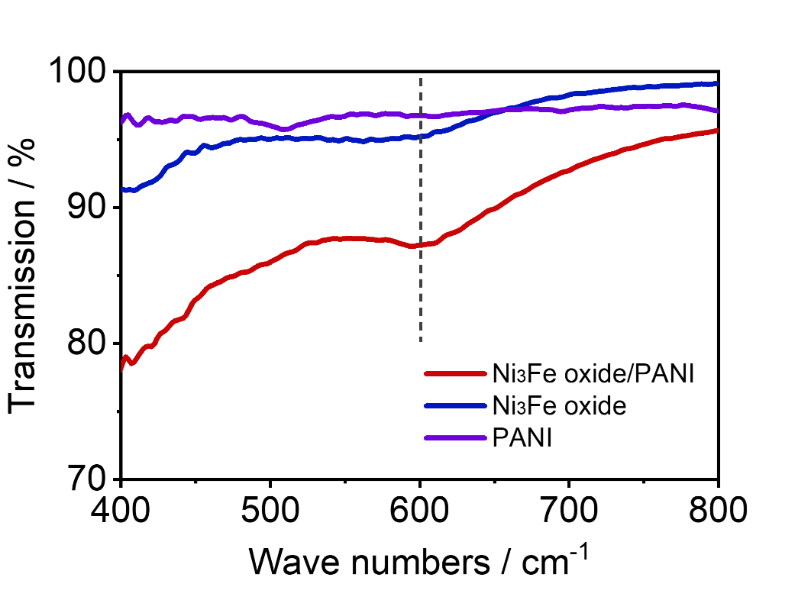


**Fig. S7** FTIR patterns for PANI, Ni_3_Fe oxide/PANI and Ni_3_Fe oxide at the range of 800-400 cm^-1^


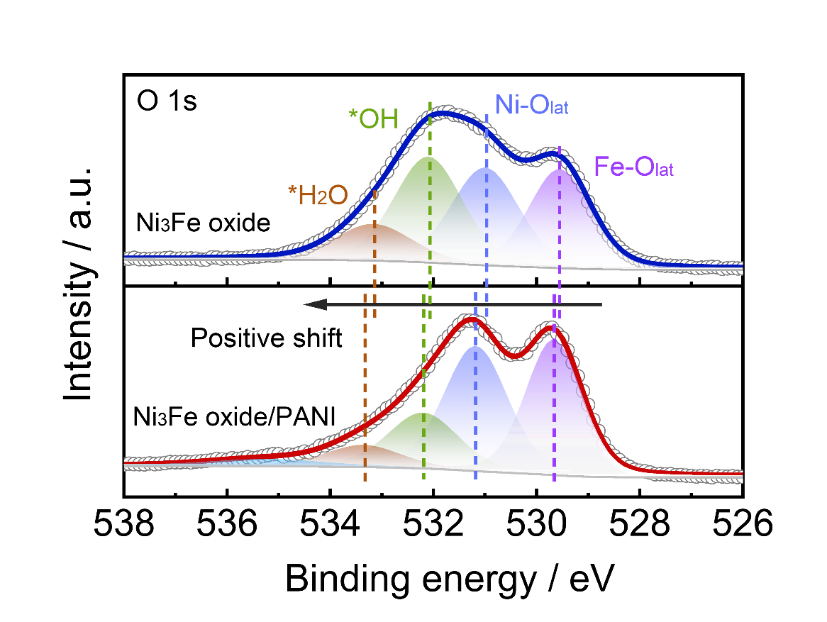


**Fig. S8** XPS spectra for the O 1s of the Ni_3_Fe oxide and Ni_3_Fe oxide/PANI catalysts


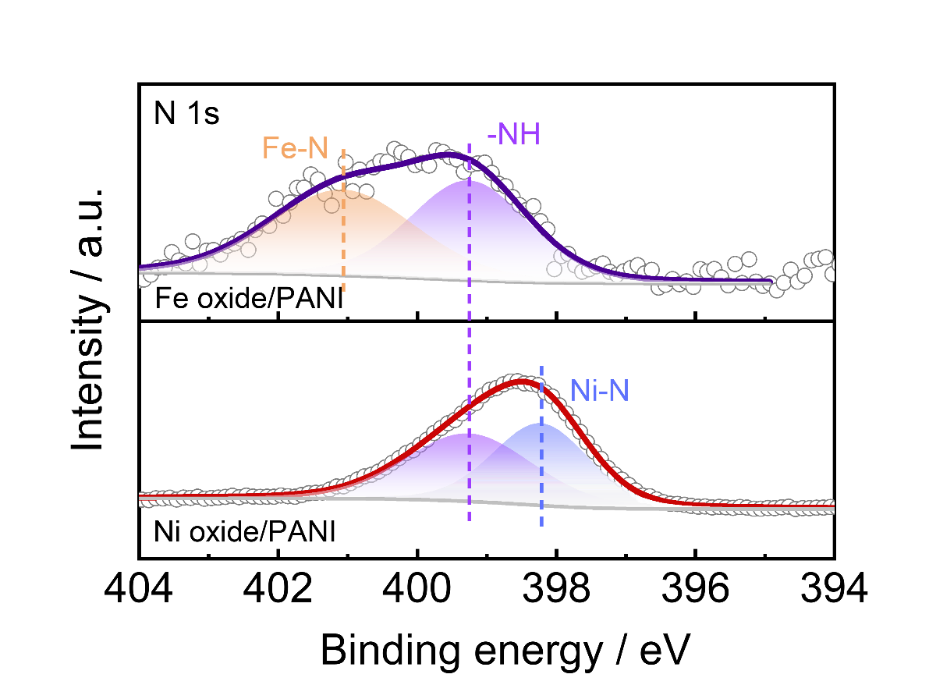


**Fig. S9** XPS spectra for the N 1s of the Fe oxide/PANI and Ni oxide/PANI catalysts


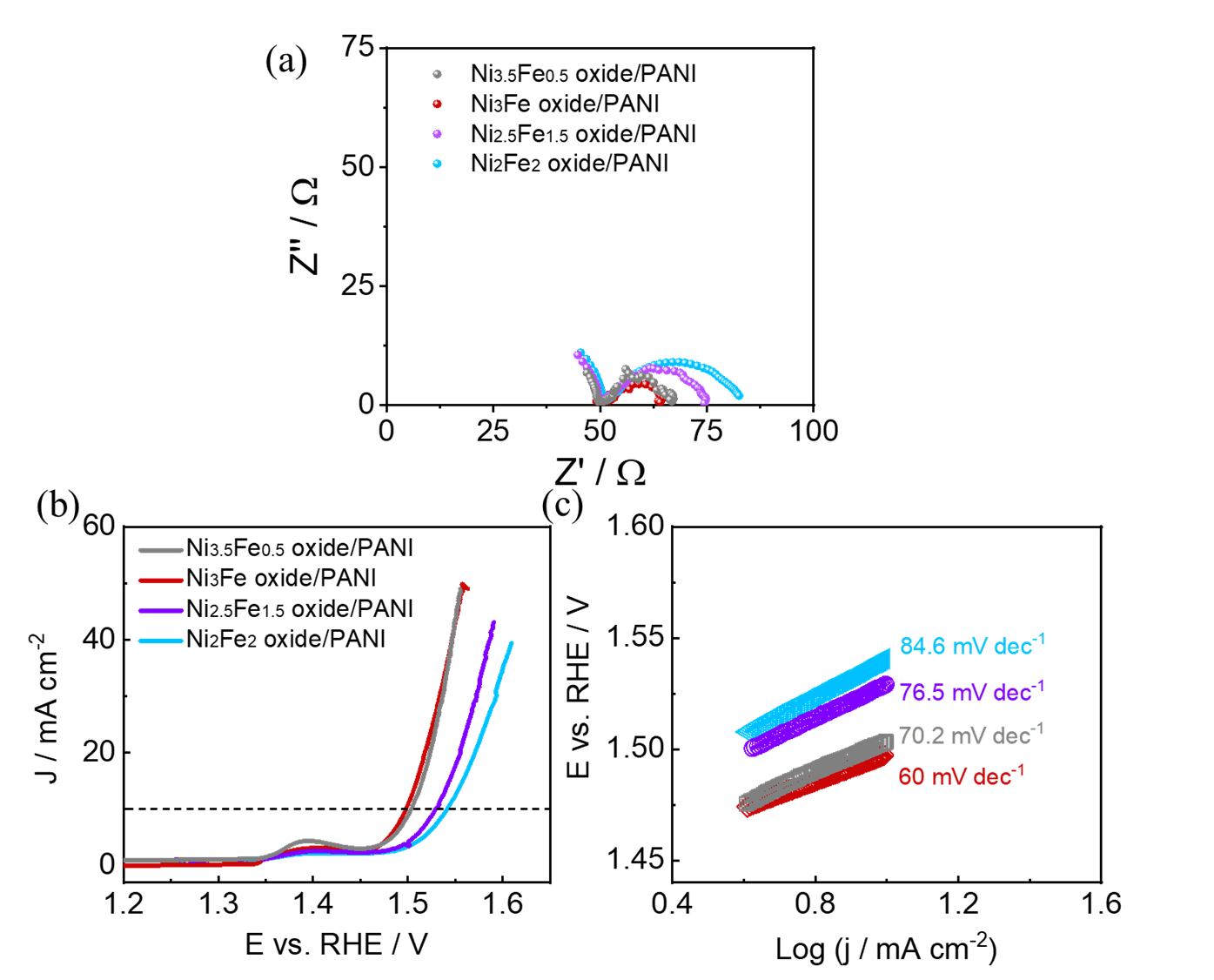


**Fig. S10** (**a**) EIS curves, (**b**) the OER LSV polarization curves, and (**c**) Tafel slopes for Ni_3_Fe oxide/PANI, Ni_2_Fe_2_ oxide/PANI, Ni_2.5_Fe_1.5_ oxide/PANI, and Ni_3.5_Fe_0.5_ oxide/PANI catalysts


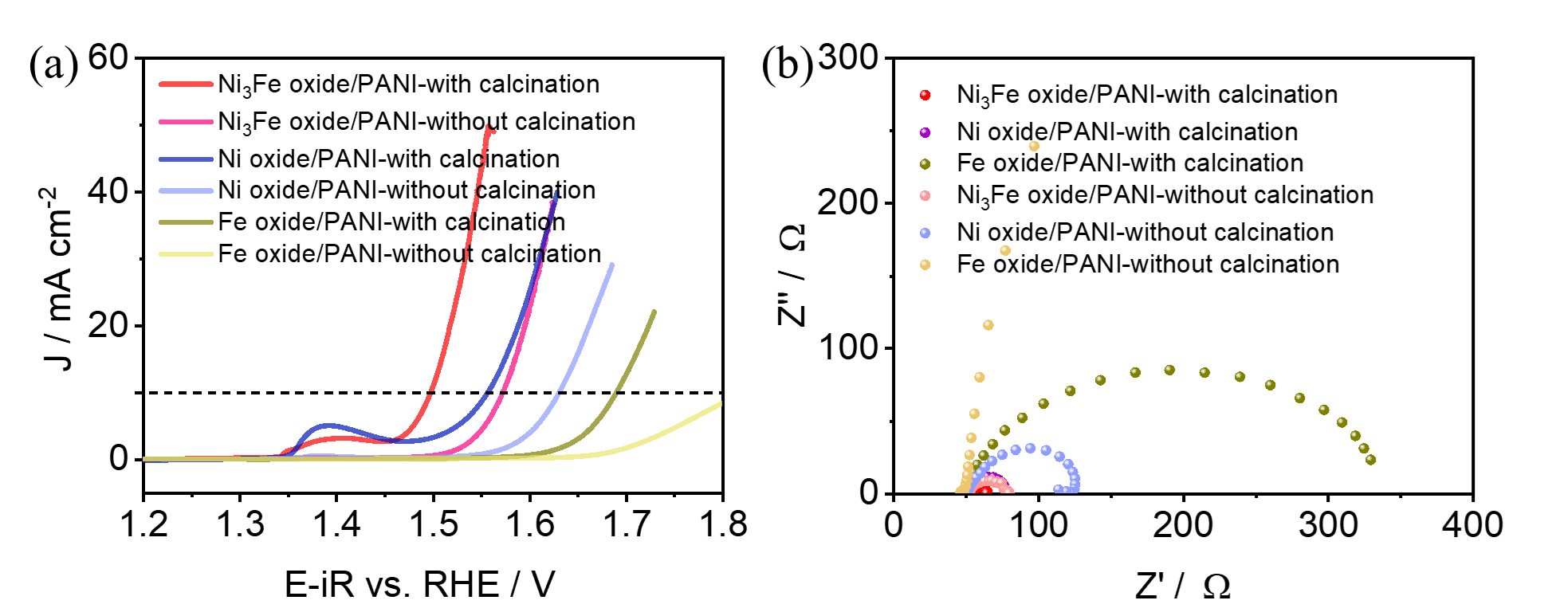


**Fig. S11** (**a**) The OER LSV polarization curve and (**b**) EIS curve for Ni_3_Fe oxide/PANI, Ni oxide/PANI, and Fe oxide/PANI catalysts with or without calcination procedures


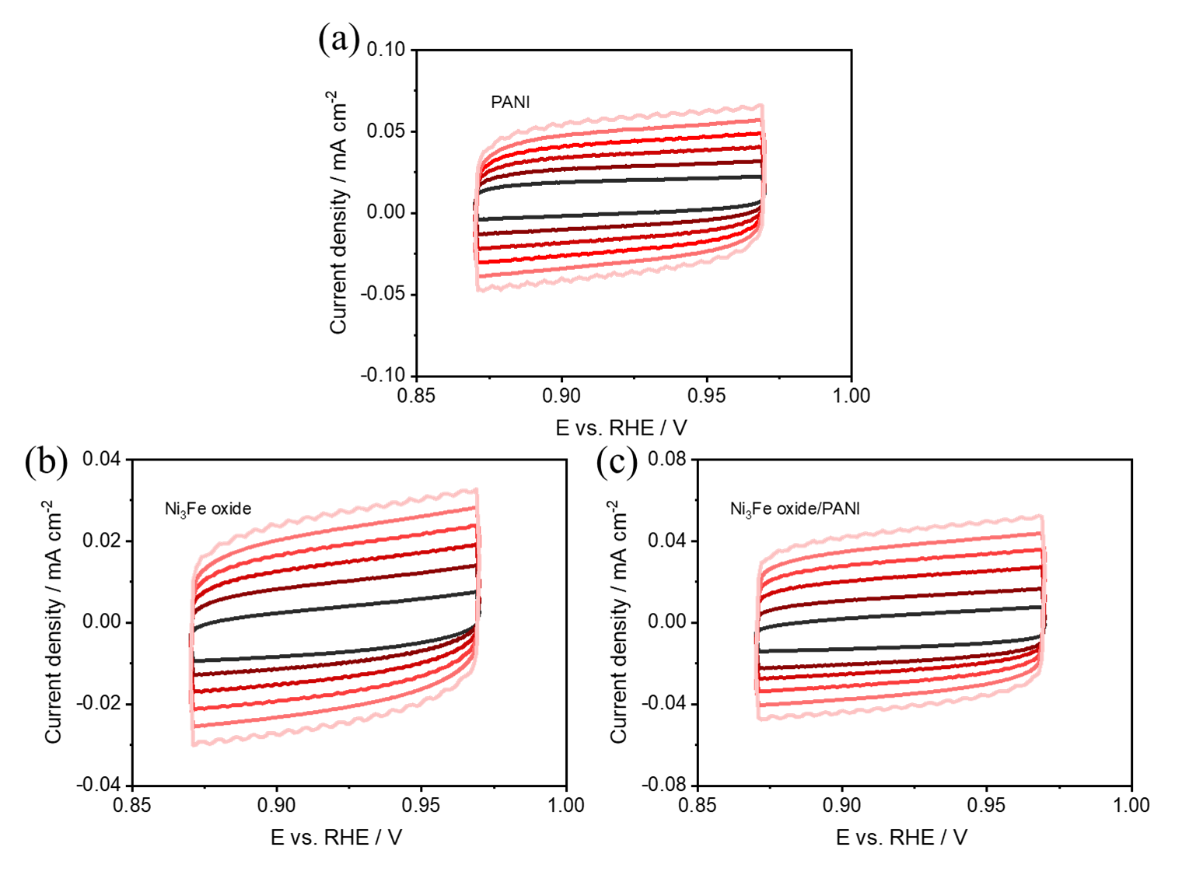


**Fig. S12** CV curves with different scan rates (10, 20, 30, 40, 50, and 60 mV s^-1^) at the range of 0.87-0.97 V vs. RHE: (**a**) PANI, (**b**) Ni_3_Fe oxide, and (**c**) Ni_3_Fe oxide/PANI


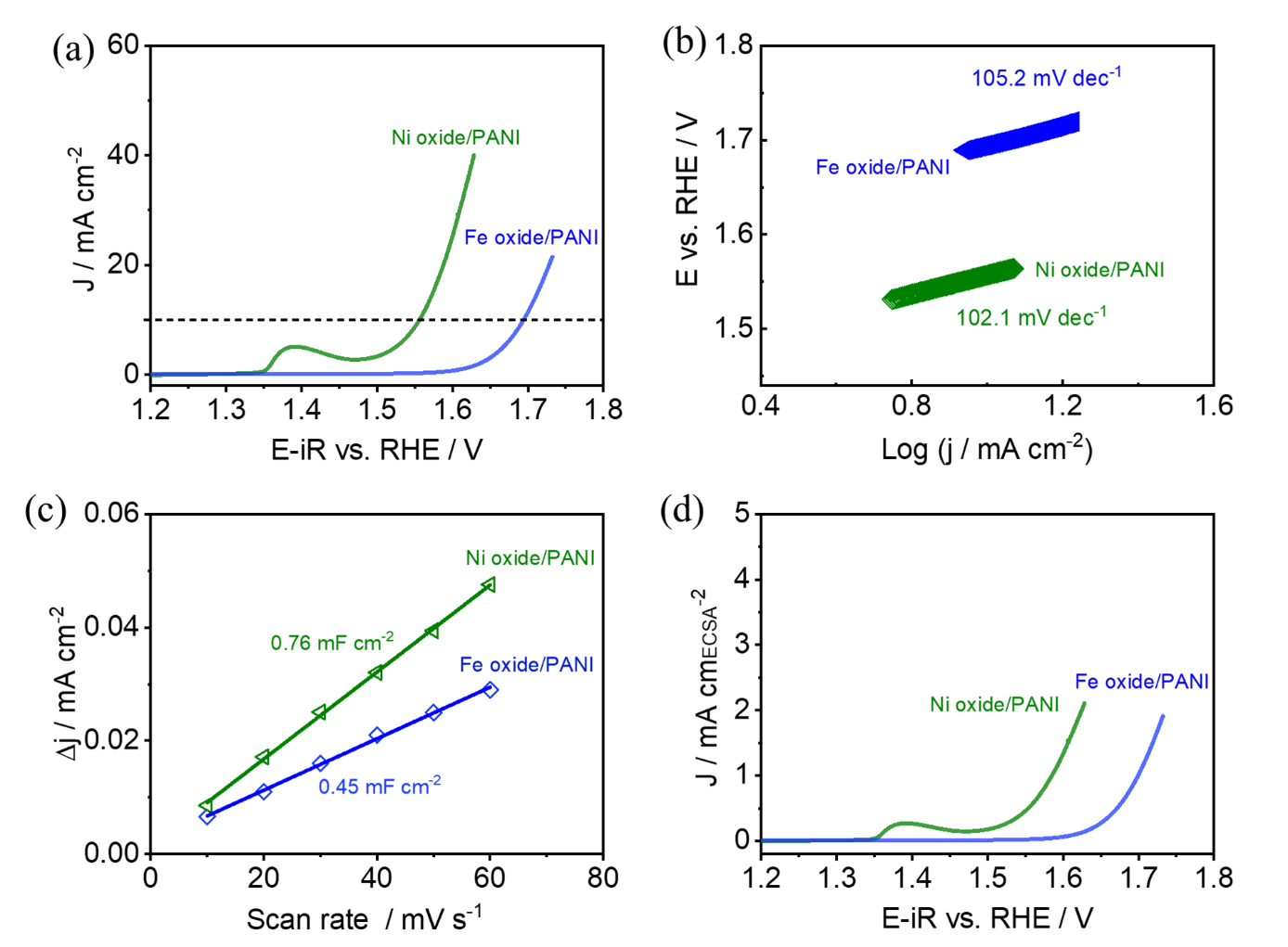


**Fig. S13** (**a**) the OER LSV polarization curves, (**b**) Tafel slopes, (**c**) Half of the difference in current density at 0.87-0.97 V (vs. RHE) versus scan rate from 10-60 mV s^-1^, and (**d**) OER activity based on the ECSA value for the Ni oxide/PANI and Fe oxide/PANI catalysts


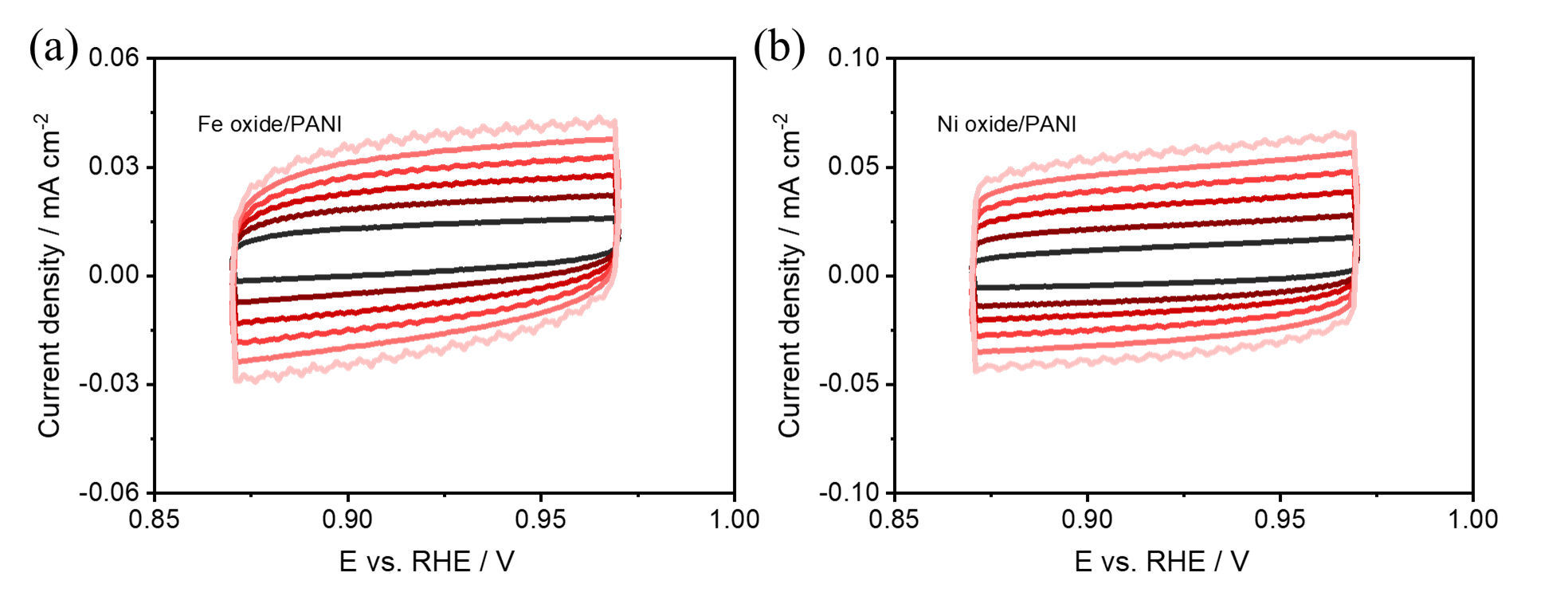


**Fig. S14** The CV curves with different scan rates (10, 20, 30, 40, 50, 60 mV s^-1^) at the range of 0.87-0.97 V vs. RHE: (**a**) Fe oxide/PANI, and (**b**) Ni oxide/PANI


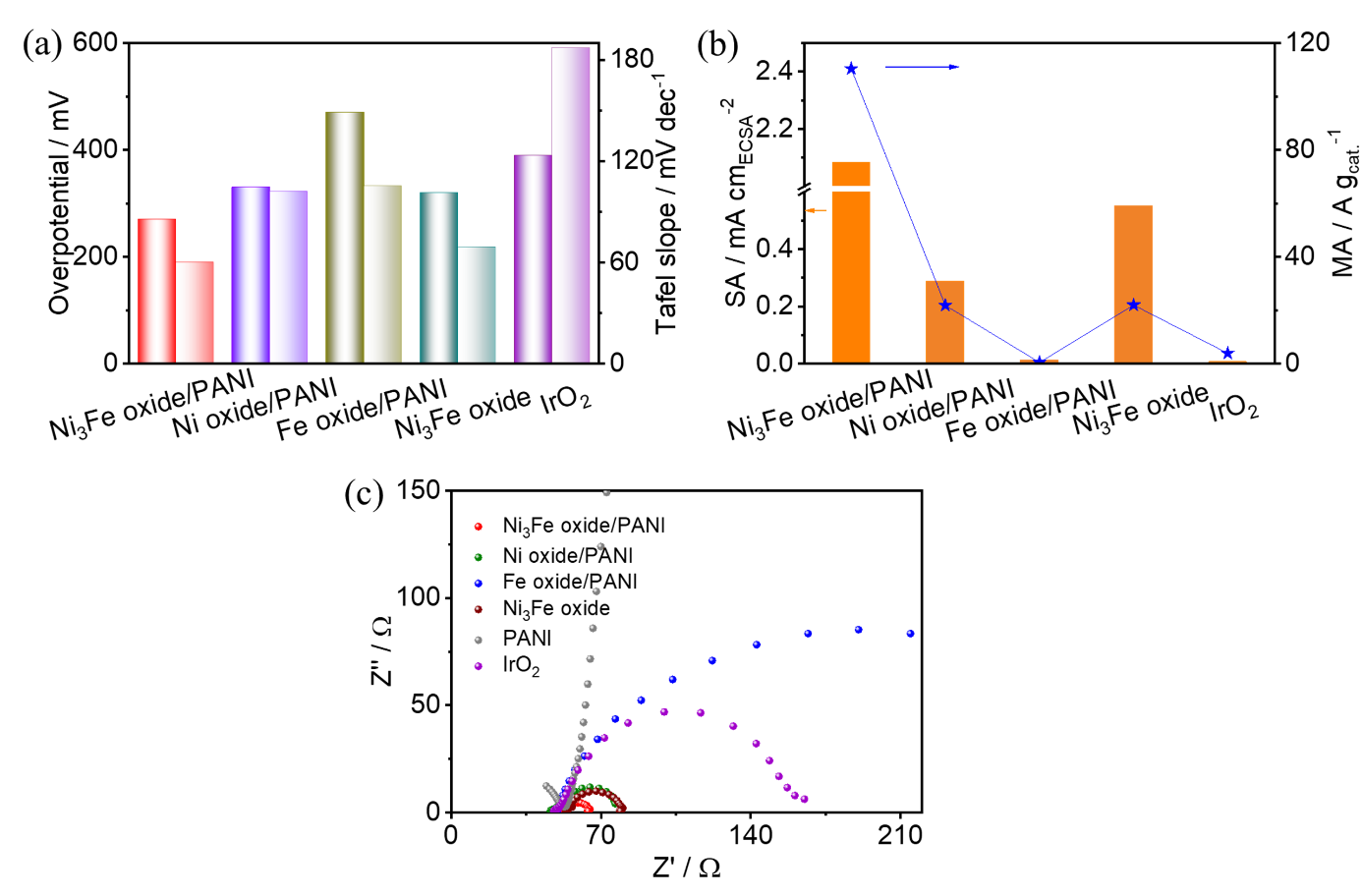


**Fig. S15** (**a**) The OER overpotential at 10 mA cm^-2^ and (**b**) the OER activity evaluation with SA and MA comparisons at overpotential of 300 mV for the Fe oxide/PANI, Ni oxide/PANI, Ni_3_Fe oxide, PANI, IrO_2_, and Ni_3_Fe oxide/PANI catalysts. (**c**) EIS spectra at the potential of 1.62 V vs. RHE for the Fe oxide/PANI, Ni oxide/PANI, Ni_3_Fe oxide, IrO_2_, and Ni_3_Fe oxide/PANI catalysts


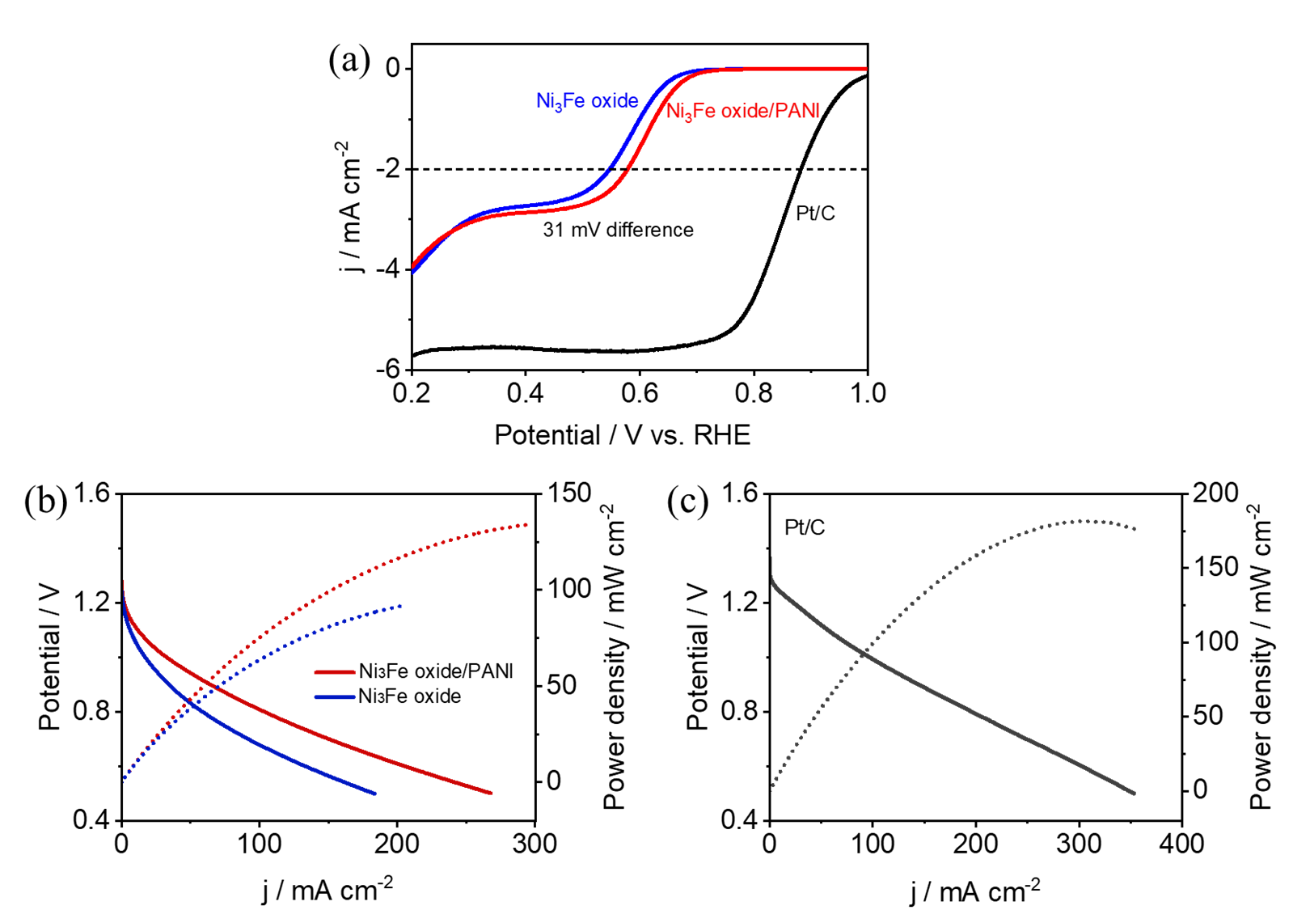


**Fig. S16** (**a**) The LSV polarization curves of Ni_3_Fe oxide, Ni_3_Fe oxide/PANI, and Pt/C catalysts at the range of 0.2-1 V vs. RHE with the scan rate of 5 mV s^-1^. (**b, c**) The discharging polarization curves with the calculated power density of Zn-air batteries with the Ni_3_Fe oxide, Ni_3_Fe oxide/PANI, and Pt/C catalysts


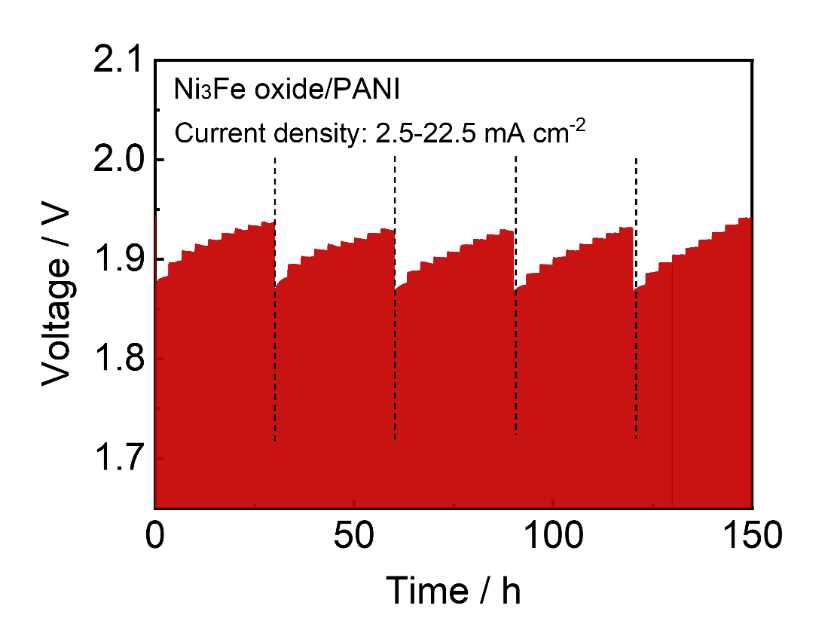


**Fig. S17**. Galvanostatic charge test at the current density from 2.5 to 22.5 mA cm^-2^ of Zn-air batteries with Ni_3_Fe oxide/PANI for five times.


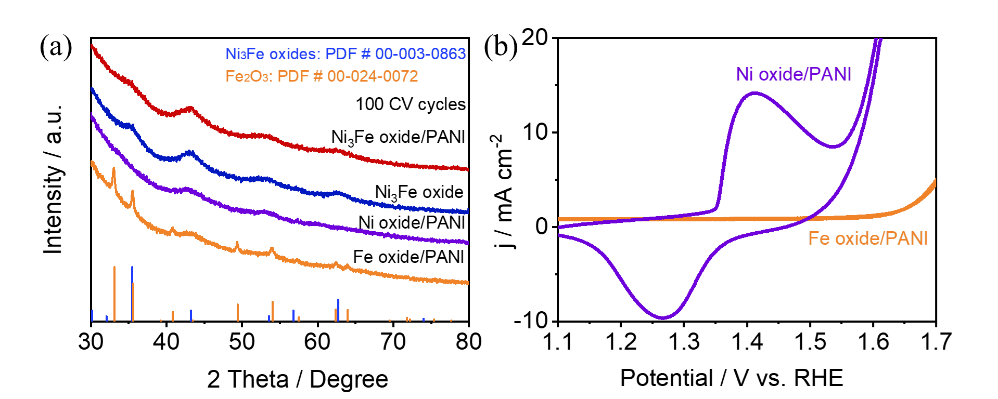


**Fig. S18** (**a**) XRD patterns after 100 CV cycles. (**b**) The CV curves for the Fe oxide/PANI and Ni oxide/PANI catalysts


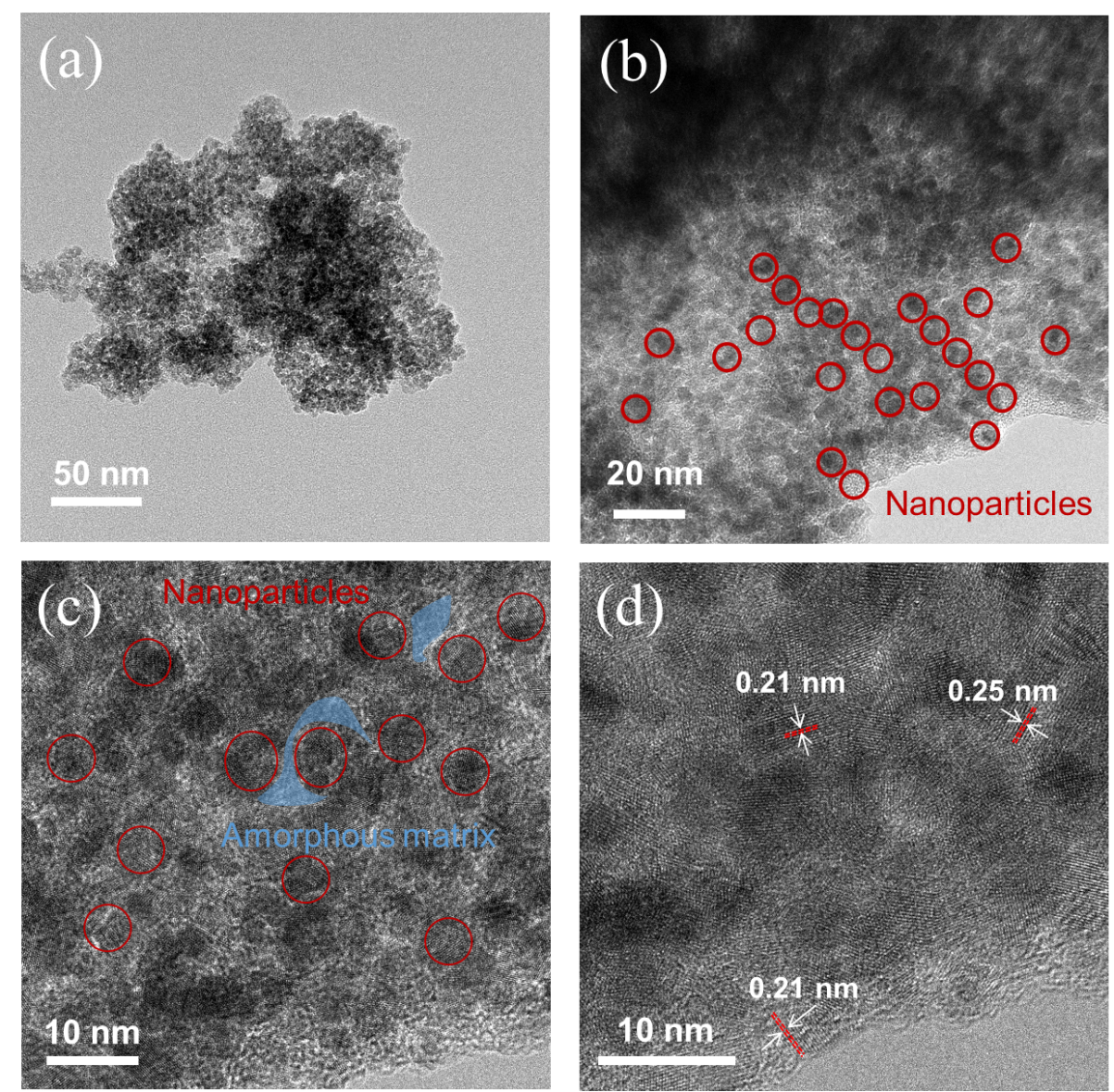


**Fig. S19** TEM images of Ni_3_Fe oxide/PANI after 10 h OER stability test


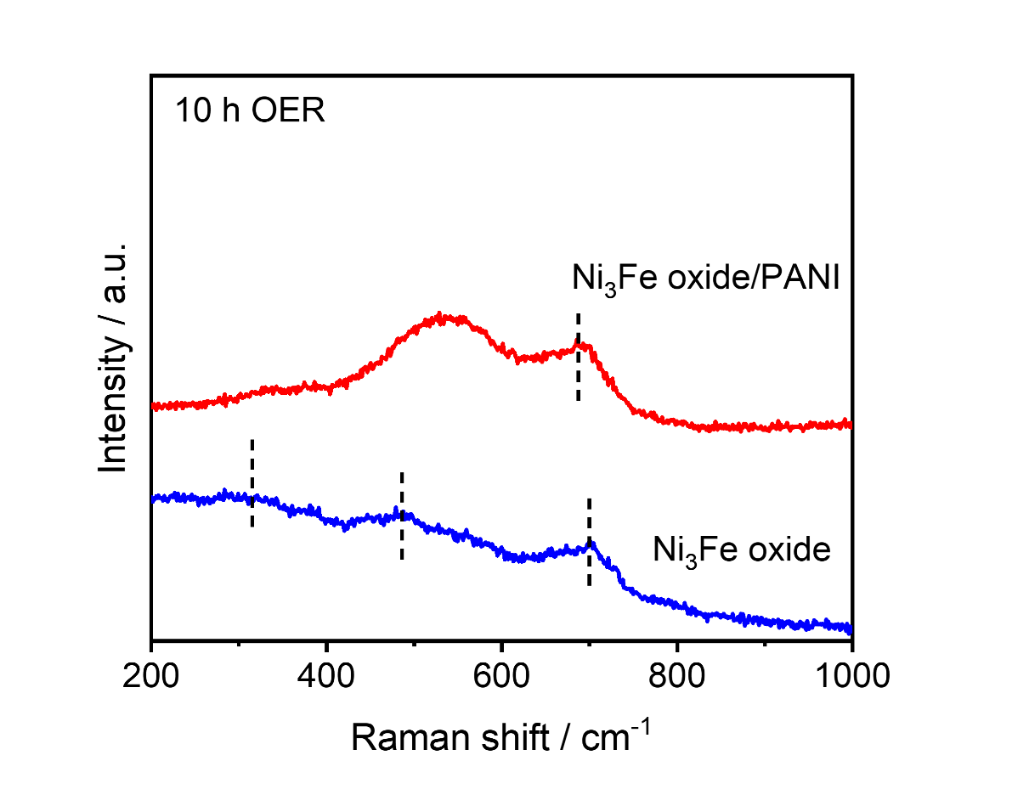


**Fig. S20** Raman spectra of Ni_3_Fe oxide and Ni_3_Fe oxide/PANI catalysts after 10 h OER stability test


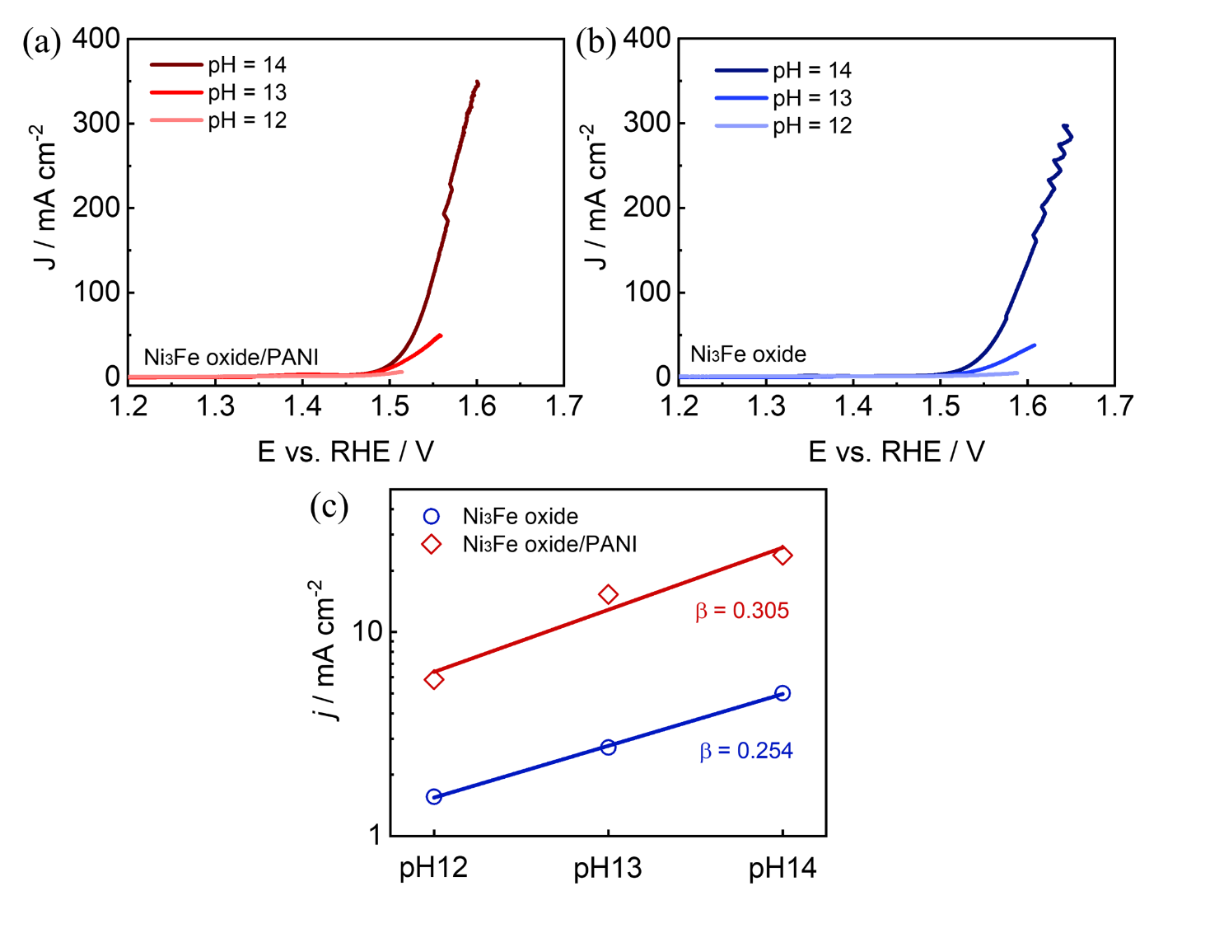


**Fig. S21.** OER LSV polarization curves at different pH conditions for (a) Ni_3_Fe oxide/PANI and (b) Ni_3_Fe oxide catalysts. (c) The reaction order parameter of Ni_3_Fe oxide/PANI and Ni_3_Fe oxide catalysts


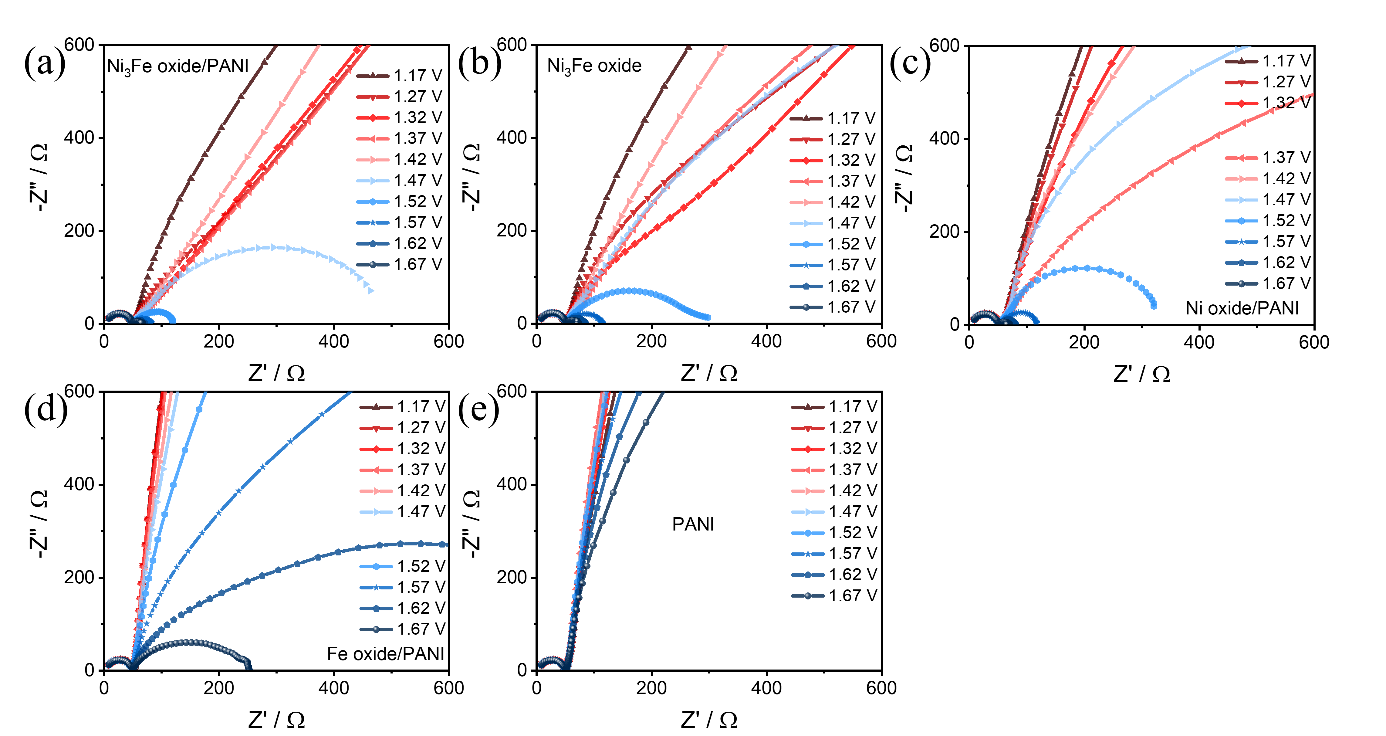


**Fig. S22** EIS spectra at the potential range of 1.17-1.67 V vs. RHE: (**a**) Ni_3_Fe oxide/PANI, (**b**) Ni_3_Fe oxide, (**c**) Ni oxide/PANI, (**d**) Fe oxide/PANI, (**e**) PANI


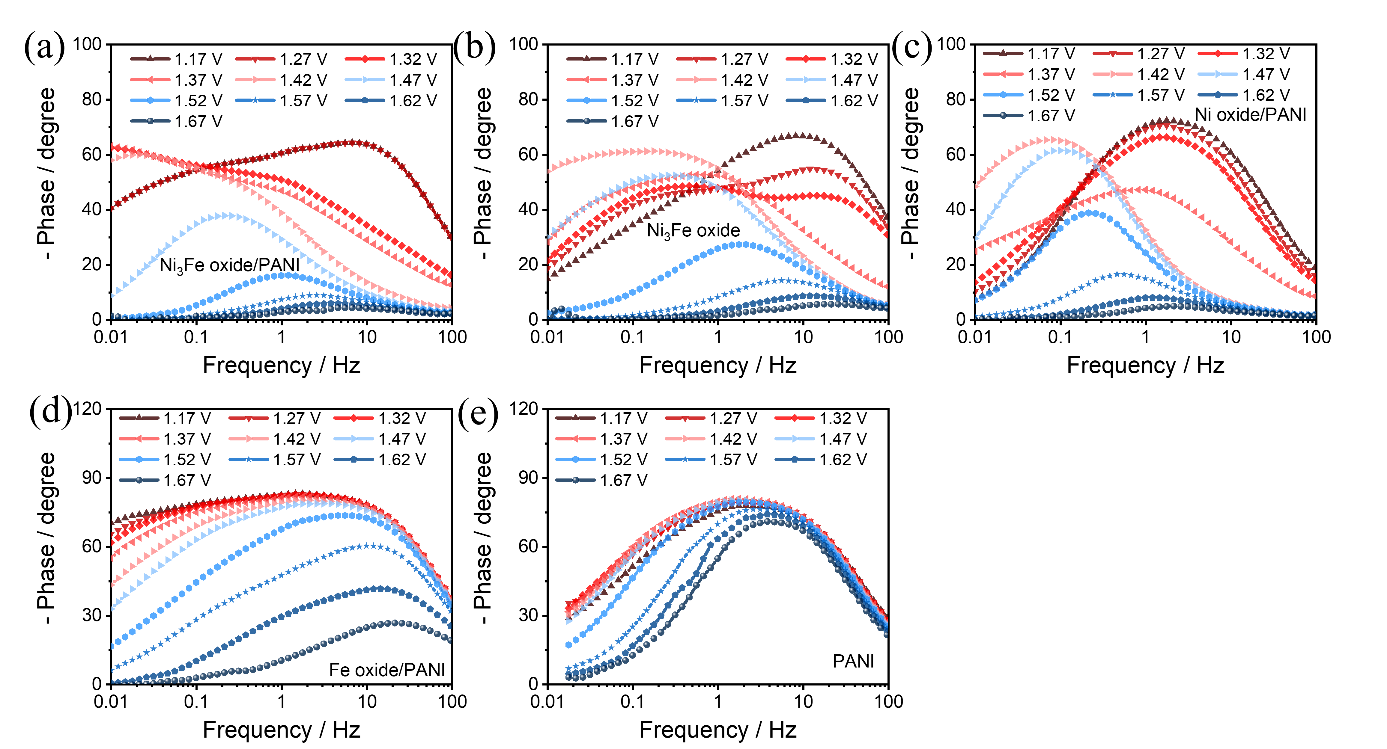


**Fig. S23** The phase value and Frequency curves at the potential range of 1.17-1.67 V vs. RHE: (**a**) Ni_3_Fe oxide/PANI, (**b**) Ni_3_Fe oxide (**c**) Ni oxide/PANI, (**d**) Fe oxide/PANI, (**e**) PANI

**Table S1** Comparison of OER activities of Ni_3_Fe/PANI catalyst with the state-of-the-art catalysts

| Catalyst | Catalyst loading  (mg cm^-2^) | Electrolyte | OER overpotential @10 mA cm^-2^ | Tafel  (mV dec^-1^) | Refs. |
| --- | --- | --- | --- | --- | --- |
| RuO_2_/CMO | 0.25-0.35 | 0.1 M KOH | 310 mV | 86 | [S1] |
| NiCo_2_O_3_@OMC | 0.567 | 1 M KOH | 280 mV | 96.8 | [S4] |
| NiFe-carbon | 0.5 | 1 M KOH | 296 mV | 56 | [S5] |
| NiFe NNG | 2 | 1 M KOH | 293.2 mV | 48 | [S6] |
| LDH/G/Ni | 2.18 | 0.1 M KOH | 325 mV | 44 | [S7] |
| FeCoNi-N-rGO | 0.06 | 0.1 M KOH | 440 mV | 124 | [S8] |
| NiFe-Mi-C-Gr | 0.5 | 0.1 M KOH | 305 mV | 90 | [S9] |
| Nd_1.5_Ba_1.5_CoFeMnO_9-δ_ | 0.418 | 0.1 M KOH | 359 mV | 81 | [S10] |
| CoS/Co_3_O_4_-5 NFs | / | 1 M KOH | 304 mV | 98.5 | [S11] |
| NiO_x_@Co_3_O_4_/CC | / | 0.1 M KOH | 360 mV | 107 | [S12] |
| Holey NCS | 0.25 | 1 M KOH | 300 mV | 53 | [S13] |
| Ni_3_FeN | 0.13 | 0.1 M KOH | 355 mV | 70 | [S14] |
| LCF-700 | 0.245 | 0.1 M KOH | 293 mV | 67 | [S15] |
| PtOaPdObNPs@Ti_3_C_2_T | 0.141 | 0.1 M KOH | 340 mV | 75 | [S16] |
| NiCoO_2_/CNTs | 0.51 | 0.1 M KOH | 420 mV | 156 | [S17] |
| RuO_2_/CeO_2_ | 0.28 | 1 M KOH | 350 mV | 74 | [S18] |
| Co@Co_3_O_4_/N-C | 0.455 | 0.1 M KOH | 390 mV | 88 | [S19] |
| N-GQDs/Co_3_O_4_ | 0.71 | 0.1 M KOH | 330 mV | 71 | [S20] |
| NiCo-LDH@HOS | 0.45 | 0.1 M KOH | 293 mV | 72 | [S21] |
| Co_3_Fe_7_@Fe_2_N/rGO | 0.71 | 0.1 M KOH | 372 mV | 114 | [S22] |
| Mn-Co_3_O_4_@CNTs | 0.136 | 0.1 M KOH | 356 mV | 68 | [S23] |
| NiFe LDH-A-Fe/NC-CNT | / | 0.1 M KOH | 360 mV | 107 | [S24] |
| Ni-CAT/NiFe-LDH/CNFs | / | 1 M KOH | 370 mV | 79 | [S25] |
| Co_9_S_8_@NiFe-LDH | / | 0.1 M KOH | 390 mV | 81.36 | [S26] |
| FeOx@N-PHCS | 0.25 | 0.1 M KOH | 340 mV | 162 | [S27] |
| LaFeO_3_ | 0.232 | 1 M KOH | 420 mV | 62 | [S28] |
| Pr_0.5_Ba_0.5_CoO_3−δ_ | 0.39 | 0.1 M KOH | 440 mV | 82 | [S29] |
| BaZr_x_Fe_1−x_O_3−δ_ | 1 | 0.1 M KOH | 412 | 97 | [S30] |
| **Ni_3_Fe oxide/PANI** | 0.25-0.35 | 0.1 M KOH | 270 mV | 60 | ***This work*** |

**Table S2** Comparison of Zn-air batteries with transition metal-based electrocatalysts

| Catalyst | Catalyst loading  (mg cm^-2^) | Power density  (mW cm^-2^) | Time (h) /  Current density  (mA cm^-2^) | Terminal cycling voltage  (V) | Refs. |
| --- | --- | --- | --- | --- | --- |
| FePc-NiCo-LDH/Ti_3_C_2_ | 1.0 | 148 | 80/10 | ~ 2.0 | [S31] |
| CoFe-NiFe/NC | / | 155 | 400/10 | ~ 1.95 | [S32] |
| Ni_1.9_FeS_1.09_(OH)_4.6_ monolith | 0.15 | 248 | 25/2 | ~2.0 | [S33] |
| NiFeS_2_/S-GO | / | 60 | 60/10 | 1.98 | [S34] |
| NiFe LDH/GQD | / | 140.04 | 120/10 | ~1.95 | [S35] |
| NiFe-MOF NSs@CQDs-COOH\|\|GO@CQDs-COOH | 1.0 | 78 | 225/5 | ~2.0 | [S36] |
| Asy-electrode | / | 236.26 | 320/10 | ~2.1 | [S37] |
| Fe2Ni@NC catalyst | 1.0 | 126 | 500/10 | ~2.0 | [S38] |
| NiFe@C@Co CNFs | / | 130 | 200/5 | ~2.1 | [S39] |
| NiFe@NGHS-NCNTs | 1.0 | 126.54 | 166/10 | ~2.2 | [S40] |
| Fe_0.5_Ni_0.5_@N-GR | 2.0 | 85 | 40/20 | ~2.0 | [S41] |
| S–LDH/NG | 0.5 | 165 | 120/5 | ~1.9 | [S42] |
| NiFe/NCNF/CC | / | 140.1 | 233/10 | ~2.0 | [S43] |
| Ni-CAT/NiFe-LDH/CNFs | 1.5 | 292.1 | 66/1 | ~2.0 | [S25] |
| Ni_3_Fe@NC-600 | 3 | 175 | 110/10 | 2.02 | [S44] |
| Ni_3_Fe oxide/PANI | 1.0 | 135 | 400/10 | 1.95 | This work |

**Supplementary References**

1. X. H. Zou, Q. Lu, J. Wu, K. E. Zhang, M. C. Tang et al., Screening Spinel Oxide Supports for RuO_2_ to Boost Bifunctional Electrocatalysts for Advanced Zn-Air Batteries. Adv. Funct. Mater. 202401134 (2024). <https://doi.org/10.1002/adfm.202401134>
2. Q. Lu, X. H. Zou, Y. F. Bu, Y. Wang, Z. P. Shao, Single-phase ruthenium-based oxide with dual-atoms induced bifunctional catalytic centers enables highly efficient rechargeable Zn-air batteries. Energy Storage Mater. **68**, 103341 (2024). <https://doi.org/10.1016/j.ensm.2024.103341>
3. Q. Lu, X. Zou, X. Wang, L. An, Z. Shao et al., Simultaneous reactant accessibility and charge transfer engineering in Co-doped RuO_2_-supported OCNT for robust rechargeable zinc-air batteries. Appl. Catal. B Environ. **325**, 122323 (2023). <https://doi.org/10.1016/j.apcatb.2022.122323>
4. Y. Zhang, X. X. Wang, F. Q. Luo, Y. Tan, L. X. Zeng et al., Rock salt type NiCo_2_O_3_ supported on ordered mesoporous carbon as a highly efficient electrocatalyst for oxygen evolution reaction. Appl. Catal. B-Environ. **256**, 117852 (2019). <https://doi.org/10.1016/j.apcatb.2019.117852>
5. R. A. Raimundo, V. D. Silva, T. R. Silva, E. S. Medeiros, D. A. Macedo et al., Synthesis and characterization of NiFe-carbon fibers by solution blow spinning and application for the oxygen evolution reaction. J. Phys. Chem. Solids **160**, 110311 (2022). <https://doi.org/10.1016/j.jpcs.2021.110311>
6. Z. Lyu, S. Yu, M. Y. Wang, P. Tieu, J. C. Zhou et al., NiFe Nanoparticle Nest Supported on Graphene as Electrocatalyst for Highly Efficient Oxygen Evolution Reaction. Small 202308278 (2023). <https://doi.org/10.1002/smll.202308278>
7. H. F. Wang, C. Tang, Q. Zhang, Towards superior oxygen evolution through graphene barriers between metal substrates and hydroxide catalysts. J. Mater. Chem. A **3**(31), 16183-16189 (2015). <https://doi.org/10.1039/c5ta03422a>
8. X. Chen, D. Chen, G. F. Li, C. Gong, Y. J. Chen et al., A hierarchical architecture of Fe/Co/Ni-doped carbon nanotubes/nanospheres grafted on graphene as advanced bifunctional electrocatalyst for Zn-Air batteries. J. Alloy Compd. **873**, 159833 (2021). <https://doi.org/10.1016/j.jallcom.2021.159833>
9. Y. Xiang, C. L. Xu, T. T. Fu, Y. B. Tang, G. J. Li et al., Enhanced bifunctional catalytic performance of nitrogen-doped carbon composite to oxygen reduction and evolution reactions with the regulation of graphene for rechargeable Zn-air batteries. Appl. Surf. Sci. **575**, 151730 (2022). <https://doi.org/10.1016/j.apsusc.2021.151730>
10. N. I. Kim, Y. J. Sa, T. S. Yoo, S. R. Choi, R. A. Afzal et al., Oxygen-deficient triple perovskites as highly active and durable bifunctional electrocatalysts for oxygen electrode reactions. Sci. Adv. **4**(6), 9360 (2018). <https://doi.org/10.1126/sciadv.aap9360>
11. J. R. Hu, Z. J. Li, D. S. Zhao, Z. Han, X. R. Wu et al., l-Lysine-induced green synthesis of CoS/Co_3_O_4_ nanoframes for efficient electrocatalytic oxygen evolution. Green Chem. **25**(18), 7309-7317 (2023). <https://doi.org/10.1039/d3gc02075d>
12. G. Y. Yang, H. Xiang, M. Rauf, H. W. Mi, X. Z. Ren et al., Plasma enhanced atomic-layer-deposited nickel oxide on Co_3_O_4_ arrays as highly active electrocatalyst for oxygen evolution reaction. J. Power Sources **481**, 228925 (2021). <https://doi.org/10.1016/j.jpowsour.2020.228925>
13. Z. W. Fang, L. L. Peng, H. F. Lv, Y. Zhu, C. S. Yan et al., Metallic Transition Metal Selenide Holey Nanosheets for Efficient Oxygen Evolution Electrocatalysis. ACS Nano **11**(9), 9550-9557 (2017). <https://doi.org/10.1021/acsnano.7b05481>
14. G. T. Fu, Z. M. Cui, Y. F. Chen, L. Xu, Y. W. Tang et al., Hierarchically mesoporous nickel-iron nitride as a cost-efficient and highly durable electrocatalyst for Zn-air battery. Nano Energy **39**, 77-85 (2017). <https://doi.org/10.1016/j.nanoen.2017.06.029>
15. S. Z. Song, J. Zhou, X. Z. Su, Y. Wang, J. Li et al., *Operando* X-ray spectroscopic tracking of self-reconstruction for anchored nanoparticles as high-performance electrocatalysts towards oxygen evolution. Energy Environ. Sci. **11**(10), 2945-2953 (2018). <https://doi.org/10.1039/c8ee00773j>
16. B. B. Cui, B. Hu, J. M. Liu, M. H. Wang, Y. P. Song et al., Solution-Plasma-Assisted Bimetallic Oxide Alloy Nanoparticles of Pt and Pd Embedded within Two-Dimensional Ti_3_C_2_T_X_ Nanosheets as Highly Active Electrocatalysts for Overall Water Splitting. ACS Appl. Mater. Interfaces **10**(28), 23858-23873 (2018). <https://doi.org/10.1021/acsami.8b06568>
17. L. Ma, H. Zhou, Y. Sun, S. L. Xin, C. H. Xiao et al., Nanosheet-structured NiCoO_2_/carbon nanotubes hybrid composite as a novel bifunctional oxygen electrocatalyst. Electrochim. Acta **252**, 338-349 (2017). <https://doi.org/10.1016/j.electacta.2017.08.192>
18. S. M. Galani, A. Mondal, D. N. Srivastava, A. B. Panda, Development of RuO_2_/CeO_2_ heterostructure as an efficient OER electrocatalyst for alkaline water splitting. Int J Hydrog. Energy **45**(37), 18635-18644 (2020). <https://doi.org/10.1016/j.ijhydene.2019.08.026>
19. Y. Wang, T. J. Hu, Y. T. Qiao, Y. Chen, L. M. Zhang, *In situ* synthesis of nitrogen doped carbon with embedded Co@Co_3_O_4_ nanoparticles as a bifunctional electrocatalyst for oxygen reduction and oxygen evolution reactions. Chem. Commun. **54**(90), 12746-12749 (2018). <https://doi.org/10.1039/c8cc07956k>
20. A. Muthurasu, S. V. S. Mers, V. Ganesh, Nitrogen doped graphene quantum dots (N-GQDs)/Co_3_O_4_ composite material as an efficient bi-functional electrocatalyst for oxygen, evolution and-oxygen reduction reactions. Int. J. Hydrog Energy **43**(9), 4726-4737 (2018). <https://doi.org/10.1016/j.ijhydene.2017.11.157>
21. K. Xiang, J. Guo, J. Xu, T. T. Qu, Y. Zhang et al., Surface sulfurization of NiCo-layered double hydroxide nanosheets enable superior and durable oxygen evolution electrocatalysis. ACS Appl. Energ Mater. **1**(8), 4040-4049 (2018). <https://doi.org/10.1021/acsaem.8b00723>
22. D. Liang, H. Z. Zhang, X. C. Ma, S. L. Liu, J. F. Mao et al., MOFs-derived core-shell Co_3_Fe_7_@Fe_2_N nanopaticles supported on rGO as high-performance bifunctional electrocatalyst for oxygen reduction and oxygen evolution reactions. Mater. Today Energy **17**, 100433 (2020). <https://doi.org/10.1016/j.mtener.2020.100433>
23. X. K. Zhang, Q. F. Liu, S. M. Liu, E. R. Wang, Manganese-doped cobalt spinel oxide as bifunctional oxygen electrocatalyst toward high-stable rechargeable Zn-air battery. Electrochim. Acta **437**, 141477 (2023). <https://doi.org/10.1016/j.electacta.2022.141477>
24. W. H. Wang, C. H. Han, W. X. Hong, Y. C. Chiu, I. H. Tseng et al., NiFe layered double hydroxide (LDH) anchored, Fe single atom and nanoparticle embedded on nitrogen-doped carbon-CNT (carbon nanotube) framework as a bifunctional catalyst for rechargeable zinc-air batteries. J. Energy Storage **85**, 111058 (2024). <https://doi.org/10.1016/j.est.2024.111058>
25. J. J. Li, Y. N. Qin, Z. T. Bai, S. F. Li, L. Li et al., Investigating the role of 3D hierarchical Ni-CAT/NiFe-LDH/CNFs in enhancing the oxygen evolution reaction and Zn-air battery performance. Appl. Surf. Sci. **648**, 159080 (2024). <https://doi.org/10.1016/j.apsusc.2023.159080>
26. Y. Zhou, J. Q. Si, H. M. Wang, X. F. Li, S. Zhang et al., Co_9_S_8_@NiFe-LDH bifunctional electrocatalysts as high-efficiency cathodes for Zn-air batteries. Energy Fuels **37**(13), 9619-9625 (2023). <https://doi.org/10.1021/acs.energyfuels.3c00938>
27. R. Hao, J. T. Ren, X. W. Lv, W. Li, Y. P. Liu et al., N -doped porous carbon hollow microspheres encapsulated with iron -based nanocomposites as advanced bifunctional catalysts for rechargeable Zn-air battery. J. Energy Chem. **49**, 14-21 (2020). <https://doi.org/10.1016/j.jechem.2020.01.007>
28. J. Dai, Y. L. Zhu, Y. J. Zhong, J. Miao, B. W. Lin et al., Enabling high and stable electrocatalytic activity of iron-based perovskite oxides for water splitting by combined bulk doping and morphology designing. Adv. Mater. Interfaces **6**(1), 201801317 (2019). <https://doi.org/10.1002/admi.201801317>
29. D. D. He, G. G. He, H. Q. Jiang, Z. K. Chen, M. H. Huang, Enhanced durability and activity of the perovskite electrocatalyst Pr_0.5_Ba_0.5_CoO_3-δ_ by Ca doping for the oxygen evolution reaction at room temperature. Chem. Commun. **53**(37), 5132-5135 (2017). <https://doi.org/10.1039/c7cc00786h>
30. K. Y. Zhu, H. Y. Liu, X. N. Li, Q. M. Li, J. H. Wang et al., Oxygen evolution reaction over Fe site of BaZr_x_Fe_1-x_O_3-δ_ perovskite oxides. Electrochim. Acta. **241**, 433-439 (2017). <https://doi.org/10.1016/j.electacta.2017.04.167>
31. G. L. Li, S. Cao, Z. F. Lu, X. Wang, Y. Yan et al., FePc nanoclusters modified NiCo layered double hydroxides in parallel with Ti_3_C_2_ MXene as a highly efficient and durable bifunctional oxygen electrocatalyst for zinc-air batteries. Appl. Surf. Sci. **591**, 153142 (2022). <https://doi.org/10.1016/j.apsusc.2022.153142>
32. X. H. Yang, H. N. Mao, Z. N. Zhou, K. R. Li, C. Li et al., Biphasic nanoalloys-based trifunctional monolith for high-performance flexible zn-air batteries and self-driven water splitting. Adv Funct Mater. 202402933 (2024). <https://doi.org/10.1002/adfm.202402933>
33. B. Wang, C. Tang, H. F. Wang, B. Q. Li, X. Y. Cui et al., Anion-regulated hydroxysulfide monoliths as OER/ORR/HER electrocatalysts and their applications in self-powered electrochemical water splitting. Small Methods **2**(12), 201800055 (2018). <https://doi.org/10.1002/smtd.201800055>
34. D. J. Zhou, Y. Jia, H. B. Yang, W. W. Xu, K. Sun et al., Boosting oxygen reaction activity by coupling sulfides for high-performance rechargeable metal-air battery. J. Mater. Chem. A **6**(42), 21162-21166 (2018). <https://doi.org/10.1039/c8ta08862d>
35. X. L. Guo, X. Q. Zheng, X. L. Hu, Q. N. Zhao, L. Li et al., Electrostatic adsorbing graphene quantum dot into nickel-based layered double hydroxides: Electron absorption/donor effects enhanced oxygen electrocatalytic activity. Nano Energy **84**, 105932 (2021). <https://doi.org/10.1016/j.nanoen.2021.105932>
36. D. Q. Song, H. Z. Guo, K. Huang, H. Y. Zhang, J. Chen et al., Carboxylated carbon quantum dot-induced binary metal-organic framework nanosheet synthesis to boost the electrocatalytic performance. Mater Today Energy **54**, 42-51 (2022). <https://doi.org/10.1016/j.mattod.2022.02.011>
37. S. X. Li, H. Zhang, H. W. Zhao, L. Tao, L. X. Li et al., An asymmetric electrode matching reversible kinetics of oxygen reaction for a rechargeable Zn-air battery. Chem. Eng. J. **482**, 148868 (2024). <https://doi.org/10.1016/j.cej.2024.148868>
38. J. B. Zhu, M. L. Xiao, G. R. Li, S. Li, J. Zhang et al., A triphasic bifunctional oxygen electrocatalyst with tunable and synergetic interfacial structure for rechargeable zn-air batteries. Adv. Energy Mater. **10**(4), 201903003 (2020). <https://doi.org/10.1002/aenm.201903003>
39. X. Chen, J. Pu, X. H. Hu, Y. C. Yao, Y. B. Dou et al., Janus hollow nanofiber with bifunctional oxygen electrocatalyst for rechargeable zn-air battery. Small **18**(16), 202200578 (2022). <https://doi.org/10.1002/smll.202200578>
40. Y. F. Ma, W. H. Chen, Z. Q. Jiang, X. N. Tian, X. Y. WangGuo et al., NiFe nanoparticles supported on N-doped graphene hollow spheres entangled with self-grown N-doped carbon nanotubes for liquid electrolyte/flexible all-solid-state rechargeable zinc-air batteries. J. Mater. Chem. A **10**(23), 12616-12631 (2022). <https://doi.org/10.1039/d2ta03110h>
41. P. T. Liu, D. Q. Gao, W. Xiao, L. Ma, K. Sun et al., Self-Powered Water-Splitting Devices by Core-Shell NiFe@N-Graphite-Based Zn-Air Batteries. Adv. Funct. Mater. **28**(14), 201706928 (2018). <https://doi.org/10.1002/adfm.201706928>
42. X. T. Han, N. N. Li, J. S. Baik, P. X. Xiong, Y. B. Kang et al., Sulfur Mismatch Substitution in Layered Double Hydroxides as Efficient Oxygen Electrocatalysts for Flexible Zinc-Air Batteries. Adv. Funct. Mater. **33**(11), 202212233 (2023). <https://doi.org/10.1002/adfm.202212233>
43. C. L. Lai, J. Y. Fang, X. P. Liu, M. X. Gong, T. H. Zhao et al., In situ coupling of NiFe nanoparticles with N-doped carbon nanofibers for Zn-air batteries driven water splitting. Appl. Catal. B-Environ. **285**, 119856 (2021). <https://doi.org/10.1016/j.apcatb.2020.119856>
44. Y. M. Liu, J. H. Ma, T. K. A. Hoang, L. J. Yang, Z. H. Chen, Well-dispersed Ni_3_Fe nanoparticles with a N-doped porous carbon shell for highly efficient rechargeable Zn-air batteries. Nanoscale **15**(3), 1172-1179 (2023). <https://doi.org/10.1039/d2nr05827h>
